# Integrated omics reveals disease-associated radial glia-like cells with epigenetically dysregulated interferon response in multiple sclerosis

**DOI:** 10.1016/j.neuron.2025.09.022

**Published:** 2025-10-10

**Authors:** Bongsoo Park, Alexandra M. Nicaise, Dimitrios Tsitsipatis, Liviu Pirvan, Daniel Zucha, Andi Munteanu, Pranathi Prasad, Miguel Larraz Lopez De Novales, Cristian Bulgaru, Rafael Kollyfas, Julia Whitten, Cory M. Willis, Luka Culig, Joseph Llewellyn, Rosana-Bristena Ionescu, Magdy Mekdad, Madalena B.C. Simões-Abade, Grzegorz Krzak, Jinshui Fan, Supriyo De, Matthew O. Ellis, Marta Suarez Cubero, Angeliki Spathopoulou, Luca Peruzzotti-Jametti, Tommaso Leonardi, Gabriel Balmus, Frank Edenhofer, Myriam Gorospe, Lukas Valihrach, Irina Mohorianu, Stefano Pluchino, Isabel Beerman

**Affiliations:** 1 Epigenetics and Stem Cell Unit, Translational Gerontology Branch, National Institute on Aging, NIH, Baltimore, MD 21224, USA; 2 Department of Clinical Neurosciences and NIHR Biomedical Research Centre, University of Cambridge, Cambridge CB2 0AH, UK; 3 Computational Biology and Genomics Core, Laboratory of Genetics and Genomics, National Institute on Aging, NIH, Baltimore, MD 21224, USA; 4 Cambridge Stem Cell Institute, University of Cambridge, Cambridge CB2 0AW, UK; 5 Laboratory of Glial Biology and Omics Technologies, Institute of Biotechnology of the Czech Academy of Sciences, 25250 Vestec, Czech Republic; 6 Department of Informatics and Chemistry, Faculty of Chemical Technology, University of Chemistry and Technology, 16000 Prague, Czech Republic; 7 Faculty of Computer Science, Alexandru Ioan University, Iasi, Romania; 8 UK Dementia Research Institute at University of Cambridge, Cambridge, UK; 9 Institute of Molecular Biology, Department of Genomics, Stem Cell Biology and Regenerative Medicine & CMBI, Leopold-Franzens-University Innsbruck, Innsbruck, Austria; 10 Department of Metabolism, Digestion and Reproduction, Imperial College London, London SW7 2AZ, UK; 11 Center for Genomic Science of IIT@SEMM, Instituto Italiano di Tecnologia (IIT), 20139 Milan, Italy; 12 Department of Molecular Neuroscience, Transylvanian Institute of Neuroscience, Cluj-Napoca, Romania; 13 These authors contributed equally; 14 Lead contact

## Abstract

Progressive multiple sclerosis (PMS) involves a persistent, maladaptive inflammatory process with numerous cellular drivers. We generated induced neural stem cells (iNSCs) from patient fibroblasts through a direct reprogramming protocol that preserved their epigenome, which revealed a PMS-specific hypomethylation of lipid metabolism and interferon (IFN) signaling genes. Single-cell multi-omics uncovered a novel, disease-associated radial glia-like cell (DARG) subpopulation in PMS cell lines exhibiting senescence and potent IFN responsiveness driven by specific transcription factors. Functionally, PMS iNSCs induced paracrine senescence and inflammation onto control cells, which was inhibited upon senolytic treatment. We identified in PMS brains a distinct population of senescent, IFN-responsive DARGs that developmentally aligned with the trajectories of iNSCs *in vitro* and spatially associated with inflammatory glia in chronically active lesions.

DARGs may sustain smoldering inflammation, unveiling a previously unrecognized cellular axis that could underpin mechanisms in neurodegeneration. This discovery offers novel insights into disease mechanisms and highlights potential therapeutic targets.

## In brief

Park, Nicaise, Tsitsipatis, and colleagues discover transcriptional and epigenetic markers in human PMS iNSCs and fibroblasts that may underlie their senescent and inflammatory state. Novel DARGs with the potential to fuel smoldering inflammation are found in chronic lesions from people with PMS.

## Graphical Abstract

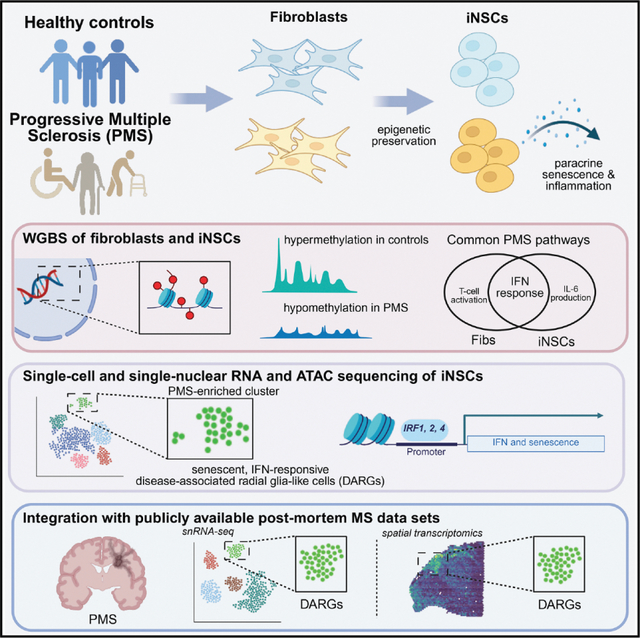

## INTRODUCTION

Multiple sclerosis (MS) is a complex neuroinflammatory and neurodegenerative disease characterized by inflammation and demyelination in the central nervous system (CNS). Its etiology is thought to be an interplay of genetic predisposition and environmental risk factors.^1^ The early phase of disease manifests as acute demyelinating lesions counteracted by some endogenous repair. Over time, this transitions to progressive MS (PMS), marked by gradual accumulation of neurological disability driven by neurodegeneration.^1^

Pathological, neuroimaging, and clinical data suggest PMS is driven by a primary smoldering process associated with inflammation involving several mechanisms, including innate immune activation, demyelination and energy deficits, adaptive immunity, and age.^2,3^

Age is a key significant risk factor for transition to PMS, similar to other neurodegenerative diseases.^4,5^ Longitudinal image assessment of brain aging demonstrated that people with MS have accelerated “brain age” with increased atrophy and reduced gray matter (GM) compared with healthy controls.^6,7^ Pathological hallmarks of cellular aging, including senescence and senescence-associated changes, have been identified in people with PMS. These include decreased telomere length in peripheral leukocytes,^8–10^ increased DNA and mitochondrial damage in neurons *in situ*,^11–13^ increased epigenetic age in glial cells,^14^ a senescence-associated secretory phenotype (SASP) in microglia and astrocytes,^15,16^ and p16^Ink4a^/*CDKN2A* expression in glia, including neural stem/progenitor cells (NSCs) and astrocytes.^16–18^ The increasing evidence linking PMS to senescence underscores the need for investigation into how the accumulation of senescent glial cells contributes to disease pathogenesis and persistent smoldering inflammation.

MS genetic risk and severity are highly complex. Genome-wide association studies (GWASs) reveal loci linked to peripheral immune cell expression, while MS susceptibility genes are enriched in CNS glial cells—astrocytes, oligodendrocytes, and microglia. Loci predicting severity relate to mitochondrial function, cellular senescence, and synaptic plasticity.^19^

Induced pluripotent stem cells (iPSCs) have enabled *in vitro* modeling of brain disorders, but limitations remain. Reprogramming resets age-related epigenetic modifications.^20–22^ Recent work shows iPSC-NSCs and directly induced NSCs from PMS patients exhibit senescence markers, hinder oligodendrocyte differentiation, and promote neuronal death via SASP.^17,23^ iPSC-derived astrocytes from MS patients show upregulation of senescence-related genes, dysfunctional metabolism, and increased inflammation.^24–26^ Thus, epigenetic age may not fully capture age-related disease processes. Environmental factors and chronic inflammation likely influence MS susceptibility and progression, as aberrant DNA methylation (DNAm) is observed in CNS tissue, blood, and leukocytes in early and PMS stages.^27,28^

Adult NSCs, or radial glia (RG)-like cells, classically reside in the mammalian subventricular zone of the lateral ventricle and dentate gyrus of the hippocampus and can differentiate into mature neurons, astrocytes, and oligodendrocytes.^29^ Noncanonical RG-like cell niches are reported in primate neocortex,^30^ rabbit cerebellum,^31^ mouse amygdala,^32^ and human striatum.^33^ Mammalian studies have shown progressing age and neurodegenerative disease diminish the capabilities of RG-like cells to differentiate or perform repair processes.^34–36^ Immunostaining of post-mortem human brains^37^ identified RG-like cells lining the lateral ventricle, the dentate gyrus, and the olfactory epithelium.^38,39^ However, their capacity for neurogenesis and existence outside of the main CNS germinal stem cell niches is debated.^40,41^ More recently, inflammation and injury have been shown to promote both the adaptive reprogramming of neurons^42^ and the de-differentiation of astrocytes^43^ into RG-like cell states. RG-like cells expressing features of senescence have also been identified in demyelinated lesioned areas in post-mortem MS brain tissue.^17,44,45^

To investigate the senescent phenotype in NSCs, we utilized direct reprogramming technology to preserve epigenetic memory of the donor cells, generating induced (i)NSCs.^46,47^ DNAm analysis on parental fibroblasts and iNSCs revealed hypomethylated genes involved in inflammatory and interferon (IFN) pathways in PMS iNSCs, indicating an epigenetic predisposition toward inflammation. Comprehensive transcriptomic profiling confirmed increased RNA expression of senescence, inflammation, and IFN signaling pathways in PMS-derived iNSCs driven by IFN-associated transcription factors (TFs). Functional studies employing senolytic treatment of PMS iNSCs revealed a significant reduction in IFN and inflammatory signaling pathways, preventing the propagation of senescence-associated transcriptional programs. Corroborating analyses of post-mortem single-nucleus and spatial transcriptomics datasets identified a distinct, non-neurogenic, disease-associated RG-like cell population (DARGs) within chronic active lesions with the potential to fuel smoldering inflammation in PMS.

## RESULTS

### Bulk RNA-seq reveals upregulation of senescence and inflammatory pathways in PMS iNSCs

**Figure 1.**
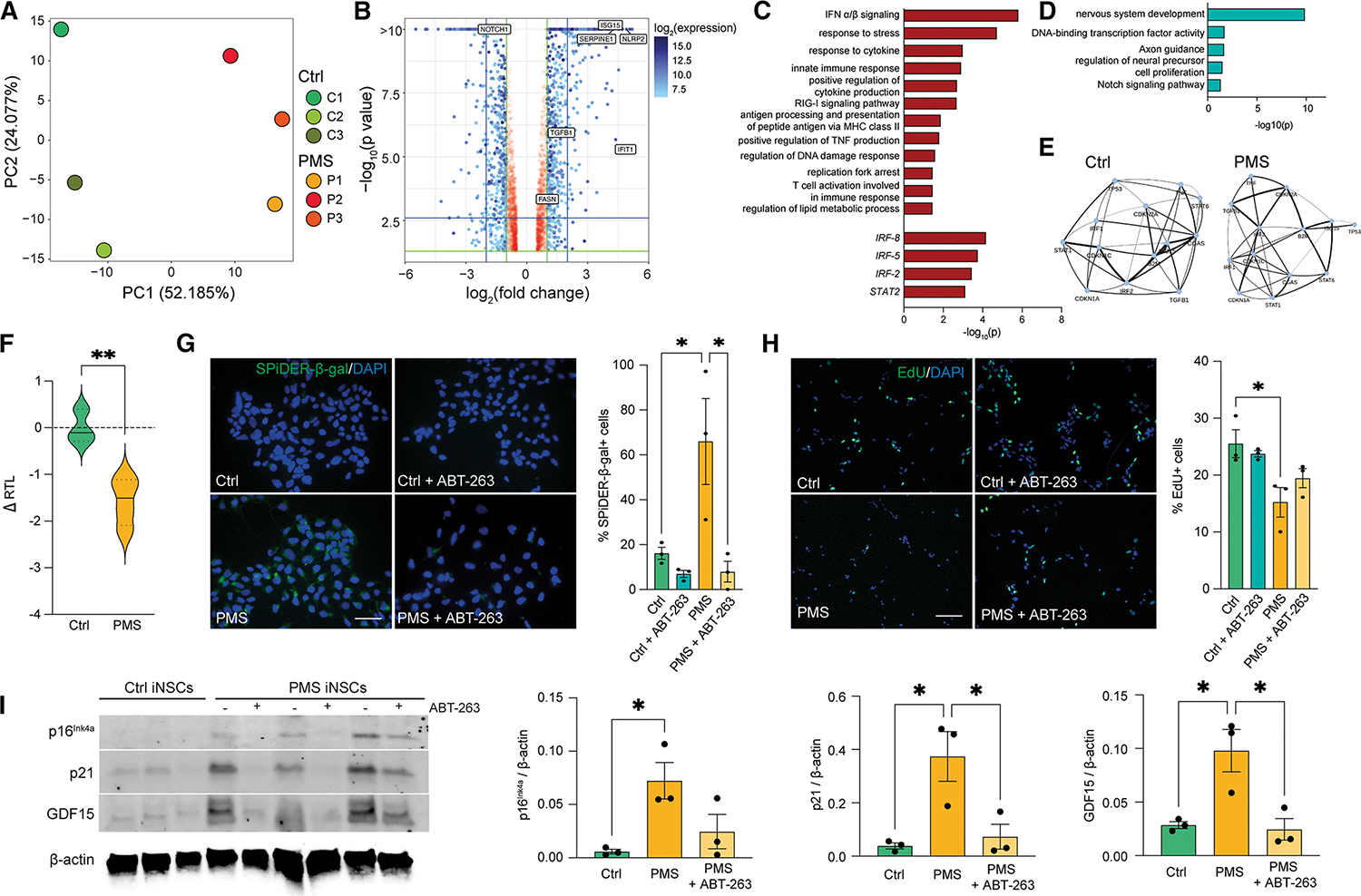
Increased inflammatory signaling and senescence markers in PMS iNSCs (A) Bulk RNA-seq PCA on the individual iNSC lines. (B) Volcano plot of differentially expressed genes vs. log_2_ abundance in PMS iNSCs compared with Ctrl iNSCs. (C and D) Pathway enrichment analysis, enriched (C) and depleted (D) on Gene Ontology (GO) and reactome (REAC) terms and TFs from bulk RNA-seq. (E) GRNs derived from the bulk RNA-seq. (F) Quantification of PMS relative telomere length (RTL) over Ctrl iNSCs. (G) Representative images and quantification of SPiDER-β-gal in control, PMS, and ABT-263-treated iNSCs. Scale bar, 50 μm. (H) Representative images and quantification of 5-ethynyl-2’-deoxyuridine (EdU) in control, PMS, and ABT-263-treated iNSCs. Scale bar, 100 μm. (I) Representative western blots and quantification for p16^Ink4a^, p21, GDF15, and β-actin in control, PMS, and ABT-263-treated iNSCs. Experiments in (F)–(I) were done on *n* = 3 Ctrl and *n* = 3 PMS iNSC lines, each performed in triplicate. Each data point represents an individual cell line. Data in (F) are mean values ± SEM. **p ≤ 0.01, unpaired *t* test. Data in (G)–(I) are mean values ± SEM. *p ≤ 0.05, one-way ANOVA, Tukey’s multiple comparisons.

To model disease- and age-associated NSC features, we directly reprogrammed skin-derived fibroblasts from healthy humans (Ctrl) and people with PMS into stably expandable iNSCs (Table S1).^48^ We confirmed expression and quantified established NSC markers and checked for the clearance of Sendai virus mRNAs (Figures S1A and S1B).^18^ Principal-component analysis (PCA) of bulk transcriptomic signatures across samples (Figure 1A) revealed a clear separation between the Ctrl and PMS samples, with quality controls (Figures S1C and S1D). Differential expression (DE) analyses highlighted transcripts related to inflammation, such as *ISG15*, *IFIT1*, and *SERPINE1* (Figure 1B). Gene set enrichment analysis (GSEA)^49^ on DE transcripts identified several pathways enriched in the PMS iNSCs, including gene sets associated with IFN-α/-β signaling (*IFIT2* and *OASL*), response to stress (*NLRP2* and *CLU*), response to cytokine (*IFIH1* and *STAT6*), lipid metabolic process (*ID2* and *ANXA1*), and TFs associated with IFN signaling (*IRF-8*, *IRF-5*, and *IRF-2*) (Figure 1C; Table S2). Gene sets with mRNAs associated with DNA-binding TF activity (*FOXO1* and *HES6*) and cell proliferation (*PROX1* and *INSM1*) were depleted in PMS iNSCs (Figure 1D). This is concordant with previous work identifying cellular senescence and inflammatory signaling in PMS-patient-derived NSCs.^17,18,23^

Next, we investigated the dynamics of gene regulatory networks (GRNs)^50^ associated with terms including inflammation, cellular senescence, and IFN signaling. We inferred a hub centered on the IFN gene *IRF2*, which strongly interacted with *CDKN1C*, *CDKN2A*, *IRF1*, and *CGAS* in PMS iNSCs (Figure 1E). Density plots of covariation of inflammatory and senescent transcripts showed greater difference in weights between Ctrl and PMS samples, with stronger covariation in the PMS samples compared with non-DE genes (Figure S1E). These results support the predicted interaction between senescence and inflammation pathways and the induction of a senescence and IFN program in PMS iNSCs.

Experimental validation of key senescence markers in PMS iNSCs, including shortened telomeres, increased senescence-associated β-galactosidase (SA-β-gal) activity, decreased cell proliferation, and elevated levels of senescence-associated proteins p16^Ink4a^, p21, and growth differentiation factor 15 (GDF15) (Figures 1F–1I, S1F, and S1G), supported transcriptional upregulation of senescence-associated pathways. Importantly, treatment with the senolytic drug ABT-263 (navitoclax)^51^ effectively reduced SA-β-gal activity, restored proliferative capacity, and lowered the expression of senescence-associated proteins (Figures 1G–1I).

### Epigenetic hallmarks of PMS in fibroblasts and iNSCs

**Figure 2.**
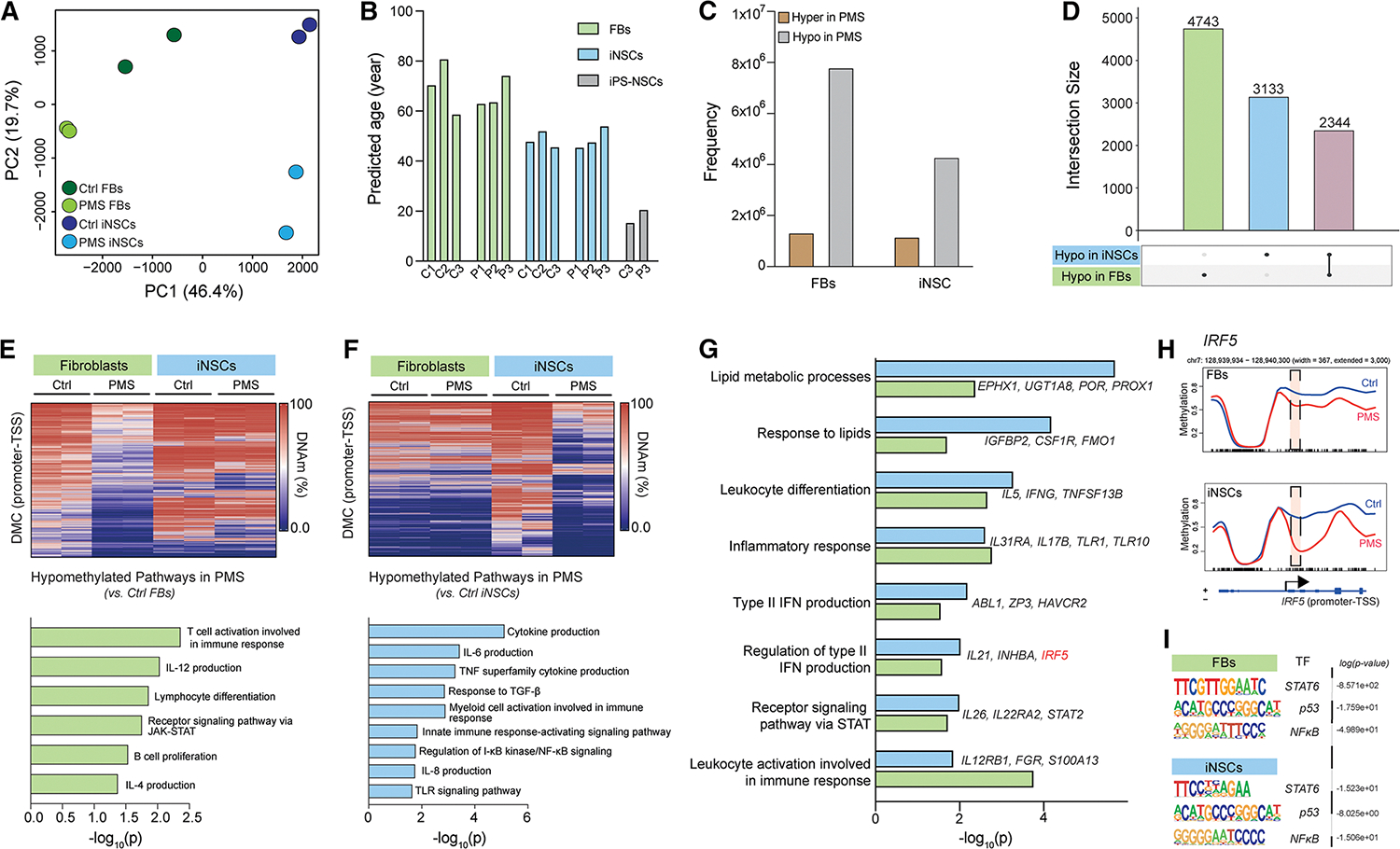
WGBS uncovers inflammatory pathways found hypomethylated in both PMS fibroblasts and iNSCs (A) PCA of WGBS data for fibroblasts and iNSC samples. (B) Cortex age DNAm aging clock inference for fibroblasts, iNSCs, and iPS-NSCs. (C) Frequency of differentially methylated cytosines (DMCs) plotted as Ctrl vs. PMS. (D) UpSet plot of hypomethylated genes within the promoter-transcription start site (TSS) region. (E and F) Heatmap and enrichment analysis of hypomethylated genes. (G) Enrichment analysis of commonly hypomethylated genes in PMS (vs. Ctrl) fibroblasts (light green) and iNSCs (light blue). (H) Example of methylation difference for *IRF5* (genome browser tracks), as in (G). (I) Proportional sequence logos on common hypergeometric optimization of motif enrichment (HOMER) motifs resulting from an enrichment analysis from (E).

To investigate the epigenetic origin of the senescent and inflammatory signatures in PMS iNSCs, we performed whole-genome bisulfite sequencing (WGBS) on the parental fibroblasts and matched iNSC lines. PCA revealed a separation between fibroblasts and iNSCs and Ctrl and PMS samples (Figure 2A).

We identified 28 million CpG sites per sample and consistently observed hypermethylation in iNSCs (vs. fibroblasts) (Figures S1H and S1I). To examine how direct reprogramming reset the aging-related epigenome, we used the cortex age DNAm aging clock,^52^ which predicted an age for directly reprogrammed iNSCs similar to the chronological age of the donor fibroblasts, unlike iPS-NSCs (Figure 2B). We additionally used Horvath and Zhang DNAm clocks to confirm this maintenance of epigenetic age following direct reprogramming (Figure S1J).^53,54^ These results support the use of direct reprogramming technology (vs. pluripotent) to maintain the epigenome of iNSCs from fibroblasts.

We next investigated overlaps in methylation patterns between Ctrl and PMS fibroblasts and iNSCs to determine what methylation differences are retained and which are newly acquired in the generation of iNSCs. We determined a quasi-uniform distribution of differentially methylated regions (DMRs) across genic and intergenic annotations, including at DMRs associated with transcription start sites (TSSs) (Figures S1K and S1L). The differentially methylated cytosines (DMCs) and DMRs indicated hypomethylation in both PMS fibroblasts and iNSCs (vs. Ctrl; Figures 2C and S1M). To examine the relationship of methylation with transcriptional and phenotypic changes, we investigated genes with hypomethylated DMRs located in proximity to TSSs (Figure 2D; Table S3).

GSEA on hypomethylated genes of the PMS fibroblasts revealed pathways associated with T cell activation, interleukin (IL)-12 production, and janus kinase (JAK)-signal transducer and activator of transcription (STAT) signaling (Figure 2E). This was supported by enriched motifs associated with TFs in the PMS fibroblasts, including *SREBP1*, which regulates T cell growth and survival, and *DDIT3*, connected to JAK-STAT signaling (Figure S1N).^55,56^ Pathways specific to hypomethylation in PMS iNSCs included cytokine production, tumor necrosis factor (TNF) superfamily cytokine production, and regulation of I-κB kinase/nuclear factor κB (NF-κB) signaling (Figure 2F). Analyses of enriched motifs in PMS iNSCs identified *STAT5* and *IRF6*, which encode proteins involved in cytokine production, the immune response, and maintenance of senescence,^57,58^ and *ARID5A*, which encodes a protein involved in the immune response by stabilizing *IL-6* mRNA (Figures 2F and S1N).^59^

GSEA on commonly hypomethylated genes (maintained between PMS fibroblasts and iNSCs) revealed pathways involved in lipid metabolism, inflammation, and IFN production (Figure 2G). These results confirm our previous findings of increased cholesterol synthesis in PMS iNSCs.^18^ Leveraging published GWAS studies, we identified genes associated with MS progression and pathology, such as leukocyte activation and differentiation, STAT signaling, and IFN production.^60–62^ We also identified *IRF5*, a known gene variant in MS,^63^ to be hypomethylated at the promoter-TSS region in both PMS fibroblasts and iNSCs (Figure 2H).

Hypermethylated genes in PMS fibroblasts (vs. Ctrl) revealed differences in pathways associated with RNA metabolic processes and cell cycle, while differentially hypermethylated genes in PMS iNSCs (vs. Ctrl) revealed pathways associated with transcription and neuronal differentiation (Figures S1O–S1Q; Table S3). The hypermethylation pattern of genes shared between PMS fibroblasts and iNSCs included DNA-templated transcription and telomerase holoenzyme complex assembly (Figure S1R).

We next identified shared binding motifs between the PMS fibroblasts and iNSCs.^64^ This identified the master TF NF-κB, which regulates the genes responsible for both the innate and adaptive immune response and is associated with induction of senescence (Figure 2I).^62,65,66^ Finally, we extended the validation to two additional cell lines (Table S1) and confirmed similar gene patterns of hypomethylation and hypermethylation, including hypomethylation of *IRF5* at the promoter-TSS region in the PMS fibroblast and iNSC line (Figures S2A–S2E).

Our data suggest that PMS pathology is strongly linked to alterations of the epigenome in PMS fibroblasts and iNSCs. Many of these epigenetic differences involve genes that regulate inflammatory, metabolic/lipid, and IFN pathways and are associated with cellular senescence.

### iNSCs possess an RG-like signature that can be identified in transcriptomic signatures from MS post-mortem human datasets

**Figure 3.**
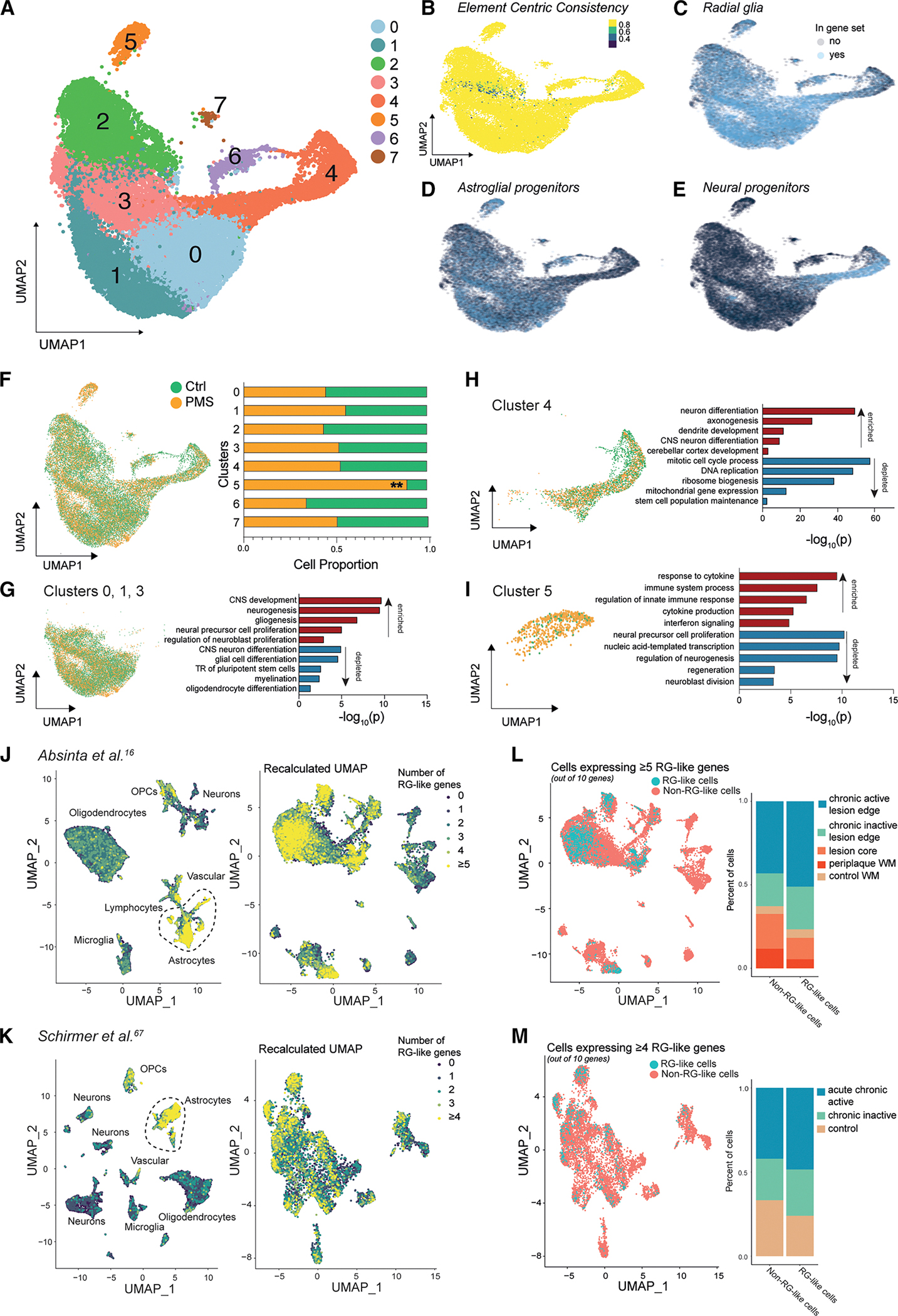
iNSCs display an RG-like transcriptomic signature that can be identified in the human MS brain using single-nuclei sequencing (A) UMAP of Ctrl and PMS iNSC scRNA-seq, snRNA-seq samples. (B) Element-centric consistency visualized on the RNA UMAP. (C–E) Voting scheme of genes associated with an RG-like (C), astroglial progenitor (D), and NPC signature (E) as in (A). (F) Cluster distribution in human iNSCs on the RNA UMAP. **p ≤ 1e−106 (χ^2^ test). (G–I) Enrichment analysis of enriched and depleted terms in clusters 0, 1, and 3 (G); cluster 4 (H); and cluster 5 (I) vs. all other clusters. (J and K) Recalculated UMAPs of RG-like cells in two *ex vivo* MS datasets, Absinta et al.^16^ (J) and Schirmer et al.^67^ (K). (L and M) Voting-scheme UMAPs and histograms summarizing the frequency of RG-like cells (and non-RG-like cells per area of interest as in J and K).

To explore the heterogeneity of iNSCs, we performed single-cell (sc) and single-nucleus (sn) RNA sequencing (RNA-seq) coupled with the assay for transposase-accessible chromatin (ATAC) with sequencing (ATAC-seq) on Ctrl and PMS iNSCs (Figure S3A). Data-driven filters were used to minimize technical discrepancies (Figures S3B–S3G), and from these data, we identified 8 clusters on the RNA modality, with high stability and with a quasi-uniform distribution of cells across all samples and conditions (Figures 3A, 3B, and S3H).

Cluster identity was assigned based on co-localized expression of standard genes for RG, astroglial progenitors, and neuronal progenitors (gene lists in Table S4). A majority of the iNSC clusters (clusters 0–3 and 5–7) were defined by an RG-gene or astroglial progenitor gene signature (Figures 3C, 3D, S3I, and S3J). A small neural progenitor cell (NPC) population was found in cluster 4 (Figures 3E and S3K). Genes associated with mature cell types, including oligodendrocyte progenitor cells (OPCs; *PDGRA*), oligodendrocytes (*OLIG1*, *OLIG2*, and *MBP*), astrocytes (*AQP4* and *ALDH1L1*), and mature neurons (*CALB* and *CCK*), were minimally expressed (Figure S3L; gene list in Table S4).^68–71^ This initial voting-scheme analysis suggests that iNSCs—similar to hiPSC-NSCs^72^—are a population of cells that display a transcriptomic signature reminiscent of RG-like cells, astroglial progenitor cells, and NPCs, with little to no detection of terminally differentiated cells.^73^

We evaluated the proportion of Ctrl and PMS cells belonging to individual clusters, and we found mostly balanced representation except for cluster 5, which was significantly enriched with PMS iNSCs (85.6% PMS vs. 14.4% Ctrl) (Figure 3F). GSEA on the cluster marker genes showed clusters 0, 1, and 3 enriched for CNS development, gliogenesis, and proliferation but depleted in cellular differentiation and pluripotency (Figure 3G; Table S5). Cluster 4 was enriched in pathways linked to neuronal and cortical development and depleted of mitotic cell-cycle genes (Figure 3H). Clusters 2, 6, and 7 were enriched for mitochondrial organization and oxidative phosphorylation (cluster 2), glial cell differentiation (cluster 6), and ion transport (cluster 7) (Figures S4A–S4C). Cluster 5 was characterized by genes enriched for cytokine production, immune processes, and IFN signaling, and genes depleted for proliferation and regeneration (Figure 3I). Cell-cycle analysis revealed increased G1-phase gene expression in cluster 4, reduced G1-phase genes in clusters 1, 2, and 3, and reduced S-phase genes in cluster 5 (Figure S4D).

To investigate the physiological relevance of the *in vitro* iNSCs to human disease, we aligned results to two independent, published *ex vivo* human snRNA-seq datasets from post-mortem MS cases and controls.^16,67^ Using canonical RG genes (Table S4), we identified RG-like cells within the annotated astrocyte clusters in both datasets (ranging between 6.5% and 7.8% of the total astrocyte cluster; Figures 3J and 3K). These cells showed higher expression of RG genes (*ETNPPL*, *PTPRZ1*, *SOX2*, *PAX6*, and *PCNA*) compared with non-RG-like cells (Figures S4E and S4F). RG-like cells expressed astroglial genes (Figures 3C and 3D) and very little microglia-specific or oligodendrocyte progenitor cell-specific genes (Figures S4E and S4F). To determine neurogenic potential of these newly identified RG-like cells, we assessed gene expression of *SOX11*, *DCX*, and *TUBB3* and found minimal to no expression in both RG-like and non-RG-like cells within the astrocyte cluster in both datasets (Figure S4G). A large proportion of the RG-like cells expressed genes specific to the G2M or S phases of the cell cycle, which indicated their ability to progress through the cell cycle (Figure S4H). RG-like cells were most prevalent in chronic active lesions (50%), whereas the smallest proportion of RG-like cells was found in control tissue (Figures 3L and 3M).

Our analyses identified a small proportion of non-neurogenic RG-like cells in healthy adult human brains, which significantly increased in frequency at chronic active lesions in PMS brains.

### PMS iNSCs harbor a senescent, IFN-responsive RG-like cell cluster reminiscent of disease-associated RG-like cells in the PMS brain

**Figure 4.**
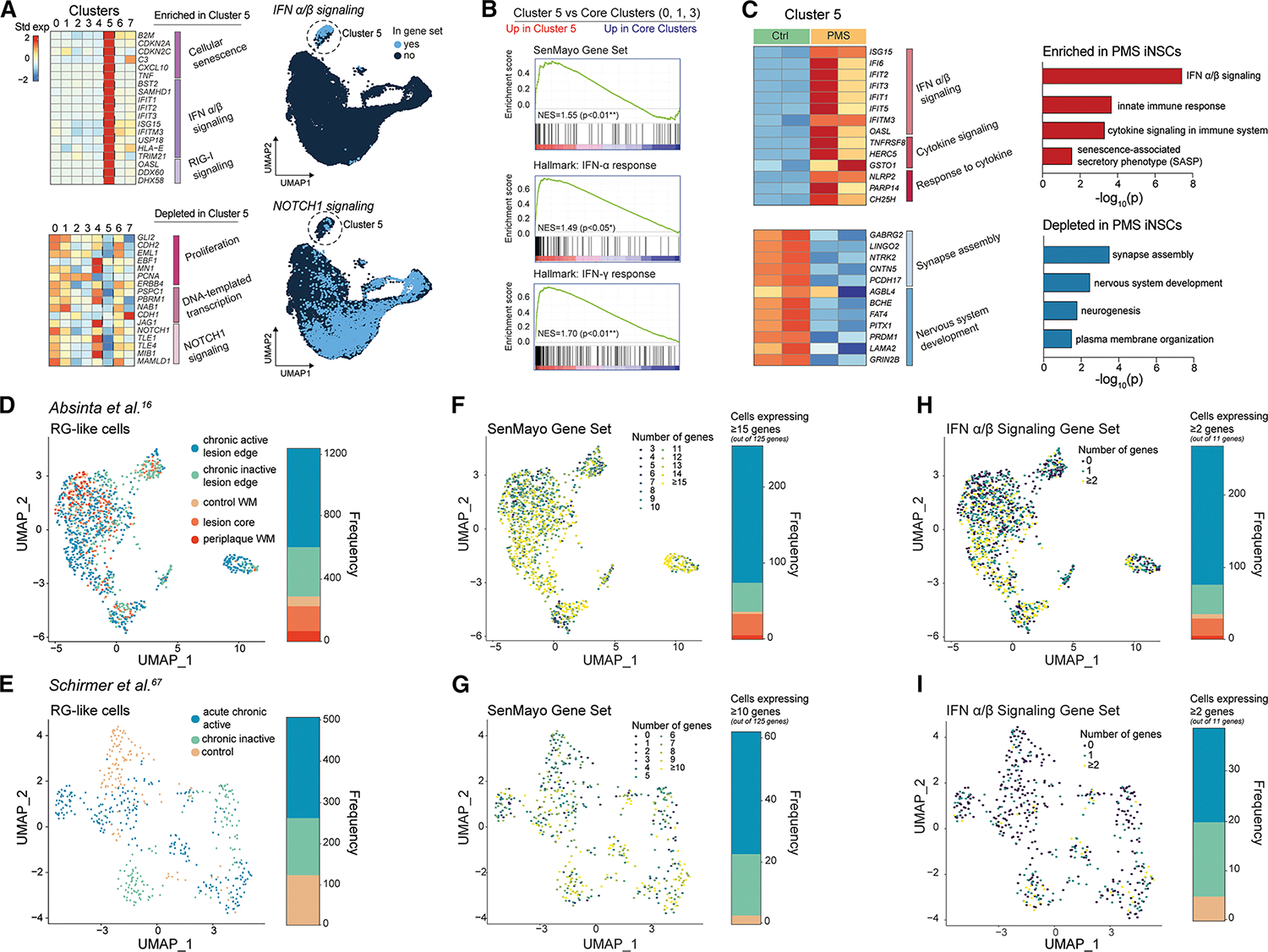
A specific PMS iNSC cluster displays senescence and IFN signaling, which is also identified in RG-like cells of the PMS brain (A) Heatmap of RNA cluster 5 signature markers and summary of associated enrichment terms. Voting-scheme UMAP of genes associated with IFN-α/-β signaling and NOTCH1 signaling. (B) Enrichment plots of the SenMayo gene set,^74^ IFN-α, and IFN-γ responses in cluster 5. (C) Heatmap of highly expressed vs. depleted transcripts in PMS vs. Ctrl iNSCs in RNA cluster 5 and GSEA. (D and E) Recalculated UMAP and histograms on the filtered cells identified as RG-like cells (Figures 3L and 3M). (F–I) UMAPs and histograms of RG-like cells that express genes in the SenMayo^74^ and IFN-α/-β signaling gene set.

Cluster 5, predominantly comprised of PMS iNSCs, showed a significant enrichment of genes associated with cellular senescence, IFN-α/-β signaling, and retinoic acid-inducible gene I (RIG-I) signaling for RNA sensing, and a significant depletion of genes associated with cell proliferation, DNA-templated transcription, and neurogenic locus notch homolog protein 1 (NOTCH1) signaling (Figure 4A). It also showed the highest expression of genes associated with IFN-α/-γ response and SenMayo genes^74^ (vs. core clusters 0–3) (Figure 4B). We performed DE analysis followed by GSEA of transcripts specific to cluster 5 and identified a strong enrichment for IFN and cytokine signaling pathways and SASP associated with high expression of *IFIT1*, *ISG15*, and *NLRP2* in PMS iNSCs (vs. Ctrl) (Figure 4C). We confirmed high expression of IFN-response genes (*IFIT1* and *IFIT2*) linked to the hypomethylated promoter regions associated with IFN signaling observed in both PMS fibroblasts and iNSCs (Figure 2H).

We next assessed SenMayo^74^ and IFN-α/β signaling gene expression in RG-like cells from the two human *ex vivo* snRNA-seq datasets^16,67^ of post-mortem MS brains (Figures 4D and 4E). A proportion (16%–28%) of total RG-like cells—which we termed disease-associated RG-like cells (DARGs)—in chronic lesions showed non-zero expression of the SenMayo gene set,^74^ whereas <5% of RG-like cells with the same features were identified in control tissues (Figures 4F, 4G, S5A, and S5B). DARGs in chronic active lesions also showed enriched IFN-associated mRNAs (Figures 4H, 4I, S5C, and S5D).

By applying the same expression thresholds to the non-RG-like cells, we determined the senescence and IFN-associated gene expression signatures are unique to DARGs. Within chronic active lesions, we identified a ~2.3-fold increase in the proportion of senescent DARGs (vs. senescent non-RG-like cells)^16,67^ (Figures S5A and S5B). Comparisons of all lesion areas identified a ~2-fold increase in the fraction of senescent DARGs in the edge of chronic active lesions when compared with the lesion core (LC), chronic inactive lesion edge, and periplaque^16,67^ (Figures S5A and S5B). DARGs in Absinta et al.^16^ showed high expression of IFN-associated genes (vs. non-RG-like cells) in chronic active lesions (Figure S5C), while displaying the same trend in chronic inactive lesions in^67^ (Figure S5D).

These findings further support the existence of non-neurogenic DARGs with an inflammatory and senescent transcription signature in the PMS brain, particularly in chronic active lesions.

### Origin of disease-associated RG-like cells from mature astrocytes in the post-mortem PMS brain

**Figure 5.**
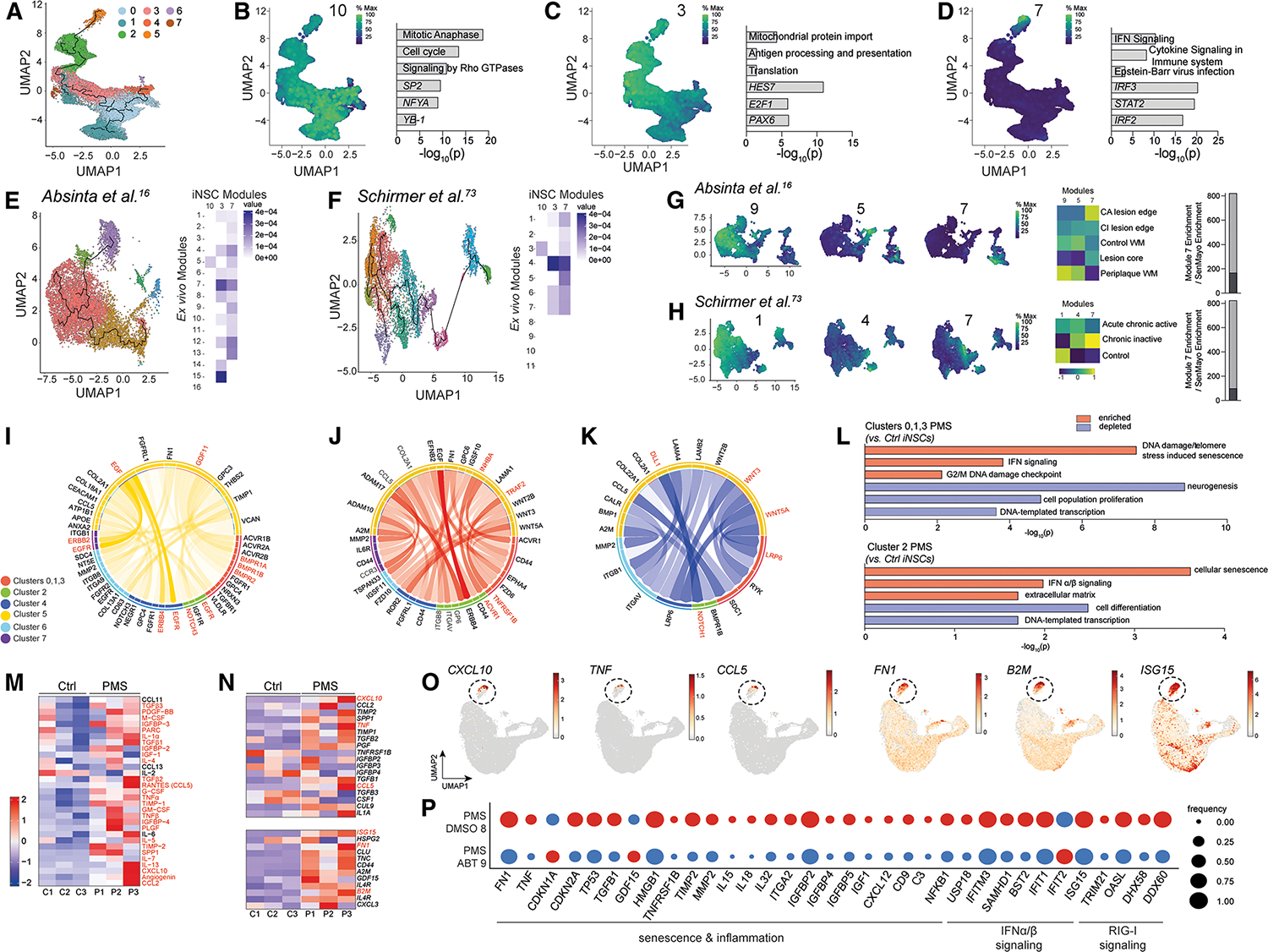
DARGs emerge from mature astrocytes, and PMS iNSCs secrete a pro-inflammatory SASP (A) Pseudotime trajectory inferred and displayed on the *in vitro* dataset UMAP. (B–D) UMAP of the distribution of intensity of genes clustered in modules 10 (B), 3 (C), and 7 (D) and GSEA and TFs corresponding to modules. (E and F) UMAP of pseudotime trajectory inferred from the Absinta et al.^16^ (E) and Schirmer et al.^67^ (F). Heatmaps summarize the scaled proportions of common genes matching between *in vitro* modules and *ex vivo* modules. (G and H) Modules selected from the *ex vivo* datasets as in (E) and (F) recapitulating the manually curated modules identified on the *in vitro* pseudotime trajectory. GSEA terms overlapping between SenMayo^74^ and module 7 for both *ex vivo* datasets. (I–K) Circos plots of the intercellular ligand-receptor interactions. (I) Yellow directed edges indicate interactions, (J) red edges summarize enriched ligand/receptor interactions, and (K) blue edges summarize depleted ligand/receptor interactions between cluster 5 and complement clusters in Ctrl and PMS iNSCs. (L) Enrichment summary on clusters 0, 1, and 3 and cluster 2 (Ctrl vs. PMS). (M) Heatmap of cytokine array performed on CM. (N) Heatmap of standardized normalized expression levels of transcripts from the bulk RNA-seq coding for secreted proteins as in (M). (O) Expression gradient UMAPs of the selected secreted proteins as genes. (P) Bubble plot of senescence, inflammation, IFN-α/-β signaling, and RIG-I signaling genes in the PMS iNSCs treated with DMSO and treated with ABT-263.

We performed pseudotime analysis to trace the developmental trajectories of the PMS iNSC DARGs (cluster 5), excluding NPCs (cluster 4), focusing on inflammatory cluster 5. We defined cluster 5 centroid as the endpoint, and the predicted pseudotime trajectory initiated at core clusters 0, 1, and 3, progressed to cluster 2, and ended in cluster 5, represented by three gene modules (Figures 5A and S5E). The expression profile of the first module (module 10) was related to the core clusters, and GSEA identified significantly elevated expression of genes associated with cell cycle and TFs known to maintain NSC/RG identity, such as *SP2* (Figure 5B; Table S6).^75^ Module 3, mainly cluster 2, was enriched for mitochondria, antigen processing and presentation, and RG-like TFs *E2F1* and *PAX6* (Figure 5C). Module 7 predominantly overlapped with cells in cluster 5 and exhibited enrichment in IFN and cytokine signaling pathways and TFs associated with IFN signaling (*IRF3* and *STAT2*) (Figure 5D). Our pseudotime analysis suggests that the progression toward cluster 5 DARGs may originate in iNSCs expressing a cluster 2-like gene signature, associated with mitochondria and cellular metabolism pathways. A metabolic signature in PMS iNSCs may promote the emergence of the newly identified IFN-responsive DARG cluster 5, which we have recently characterized.^18^

Using the gene modules from the *in vitro* dataset, we next analyzed astrocytes and RG-like cells in the *ex vivo* unifold manifold approximation and projections (UMAPs) (Figures 3J and 3K).^16,67^ We inferred pseudotime trajectories on both datasets using the module of the core *in vitro* iNSCs (clusters 0, 1, and 3) as an initialization point in the astrocyte cluster and generated new modules (Figures 5E, 5F, and S5E–S5G; Table S6). For the Absinta et al.^16^ dataset we found coordinated gene expression within modules 5 and 7 that matched our *in vitro* curated modules (3 and 7), with *ex vivo* module 7 matching the inflammatory, senescent cluster 5 (Figures 5E and 5G). Module 7 was found to be most associated with DARGs found in the chronic active lesion edge, also expressing SenMayo^74^ enrichment terms (Figure 5G). In the Schirmer et al.^67^ dataset we found coordinated gene expression within *ex vivo* modules 4 and 7 (Figures 5F and 5H). Module 7, which correlated the most with DARGs, was associated with chronic inactive lesions and SenMayo^74^ enrichment (Figure 5H). This analysis identifies the emergence of DARGs from mature astrocytes in the post-mortem MS brain.

### Cell-cell interactions predict propagation of inflammation and senescence by cluster 5

Cluster 5 RG-like cells were analyzed for senescence-associated proteins using NicheNet with cluster 5 as the source of ligands and other clusters (source of receptors).^76^ Ctrl iNSCs were enriched for ligand-receptor interactions regulating cell maintenance and differentiation (i.e., Notch signaling) in all clusters (Figure 5I).^77^ In PMS iNSCs, modeling predicted strong interactions between TNF receptor-associated factor 2 (TRAF2) in cluster 5 with TNF receptor superfamily member 1B (TNFRSF1B) in core clusters (0, 1, and 3). This suggests the induction of NFκB activation^78^ and inhibition of NSC proliferation^79^ with a depletion in Wnt signaling via low-density lipoprotein receptor-related protein 6 (LRP6) (Figures 5J and 5K). GSEA on core clusters comparing Ctrl vs. PMS iNSCs identified that PMS iNSCs were enriched for senescence pathways (e.g., DNA damage) and depleted for cell-cycle-related terms (Figure 5L). We detected significant interactions between *INHBA* from cluster 5 and *ACVR1* in cluster 2, coupled with a depletion in NOTCH1 signaling, both pathways known to be involved in regulating senescence (Figures 5J and 5K).^80,81^ Cluster 2 PMS iNSCs showed enrichment in senescence terms (e.g., IFN signaling and extracellular matrix [ECM]) and a depletion in differentiation and DNA transcription, known to be regulated by NOTCH signaling (Figure 5L).^82^ Clusters 4, 6, and 7 exhibited enrichment of inflammatory-associated terms and depletion in terms associated with NSC maintenance in PMS iNSCs (Figure S5H). The enrichment in inflammatory terms in PMS iNSCs was linked to increased inflammatory interactions between C-C chemokine receptor type 3 (CCR3) and chemokine ligand 5 (CCL5) and between alpha-2-macroglobulin (A2M) and matrix metalloproteinase-2 (MMP2), originated from cluster 5 (Figure 5J).

To further characterize the predicted ligand-receptor interactions, we performed a cytokine array on the conditioned media (CM) from the iNSC lines. The cytokine array confirmed increased secretion of inflammatory factors associated with the SASP^83^ in PMS iNSCs (vs. Ctrl) (Figure 5M), validated by bulk mRNA-seq (Figure 5N). Mapping inflammatory cytokines onto the scRNA-seq data confirmed expression in cluster 5 (Figure 5O). PMS iNSCs secrete SASP-related inflammatory factors, which may induce a dysfunctional, senescent phenotype in other clusters.

### Senolytic treatment attenuates the SASP and modulates senescence and IFN signaling in PMS iNSCs

To determine if the senescent, inflammatory cluster 5 cells are modifiable, we treated two independent cell lines (C3 and P3, Table S1) with ABT-263 and performed scRNA-seq (Figure S6A). Following quality control, most clusters exhibited an RG-like signature, with a small cluster of NPCs and minimal expression of OPC and mature cell types (Figures S6B–S6E). We identified 13 clusters in Ctrl iNSCs + DMSO or ABT-263, 9 clusters in PMS iNSCs + DMSO, and 11 clusters in PMS iNSCs + ABT-263 (Figures S6F and S6G). Clusters enriched in Ctrl + DMSO vs. Ctrl + ABT-263 (0, 2, 4, and 5) correlated with cell-cycle phases (G1/S) and were linked to processes such as nervous system development, Rho guanosine triphosphate hydrolase (GTPase) signaling, and neuron differentiation (Figures S6H and S6I). Clusters enriched in Ctrl + ABT-263 vs. Ctrl + DMSO (6 and 11) were associated with cell death, endoplasmic reticulum (ER) stress, and cell communication (Figure S6I). In the PMS iNSCs + DMSO enriched clusters (1, 2, 3, and 6), we observed enrichment for cholesterol biosynthesis, oxidative phosphorylation, and neurodegeneration markers. Whereas a new set of clusters (0, 1, 2, 3, 6, and 8) emerged after ABT-263 treatment that were linked to apoptosis, ER stress, and DNA replication (Figures S6G and S6J). While the inflammatory, senescent cluster persisted post-treatment, ABT-263 significantly reduced the expression of transcripts involved in senescence, inflammation, IFN-α/-β, and RIG-I signaling for RNA sensing, suggesting instead a senomorphic effect (Figure 5P).

**Figure 6.**
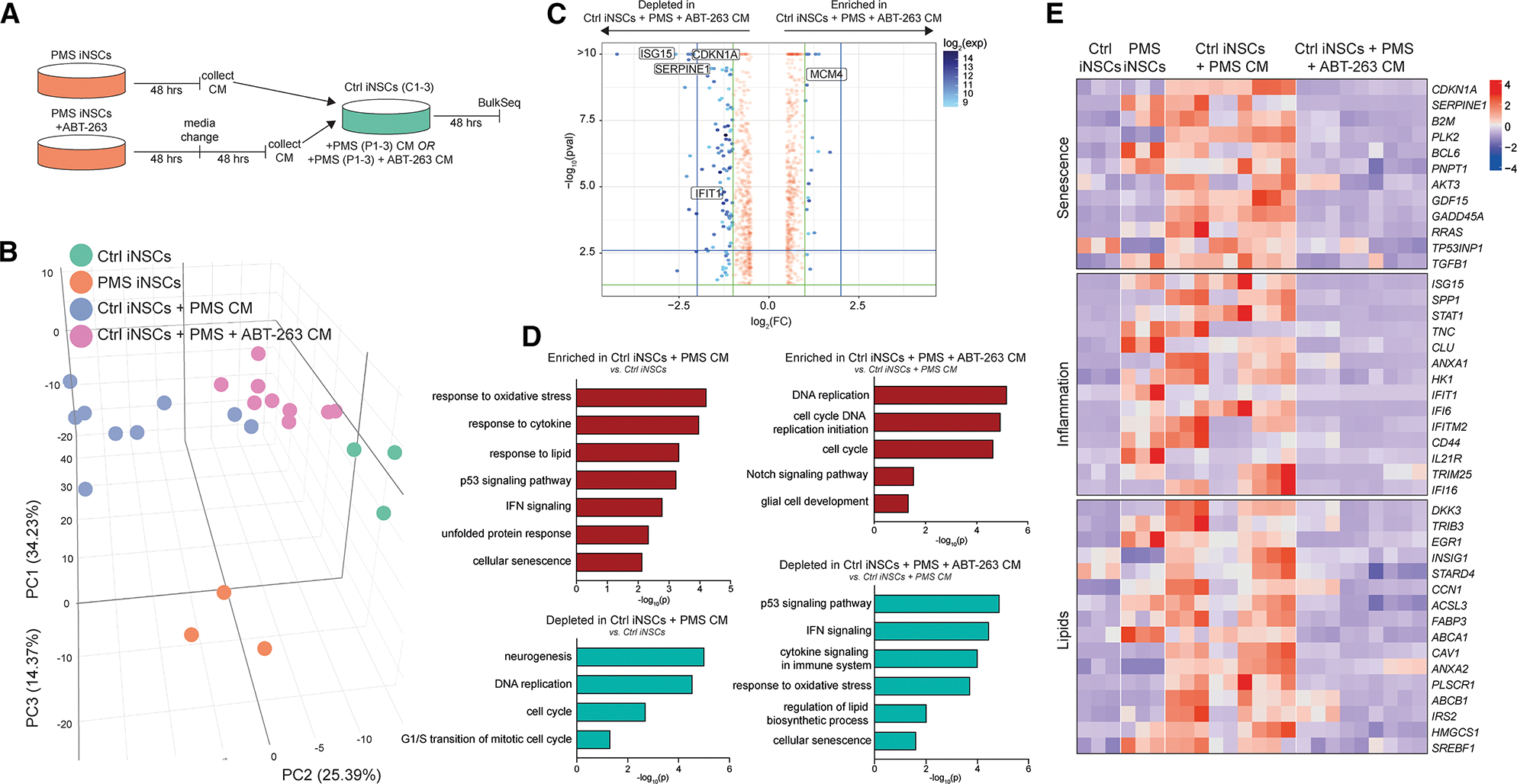
Treatment of PMS iNSCs with the senolytic ABT-263 alters the secretory phenotype (A) Experimental design of the RNA-seq conditioned media (CM) experiment. (B) PCA on the RNA-seq data of individual iNSC lines and Ctrl iNSC lines treated with PMS CM and PMS + ABT-263 CM. (C) Volcano plot of differentially expressed genes (DEGs) vs. log_2_ abundance in Ctrl iNSCs + PMS + ABT-263 CM vs. Ctrl iNSCs + PMS CM. (D) Pathway enrichment analysis on GO, REAC, and Kyoto Encyclopedia of Genes and Genomes (KEGG) terms from RNA-seq. (E) Heatmap of selected genes pertaining to senescence, inflammation, and lipids.

To assess the functional impact of the PMS-associated SASP, we treated PMS iNSCs with or without ABT-263, collected the CM, and treated Ctrl iNSCs with the CM and subsequently performed RNA-seq (Figure 6A). PCA revealed a separation between Ctrl iNSCs + PMS CM and Ctrl iNSCs + PMS + ABT-263 CM or Ctrl iNSCs without CM treatment (Figure 6B). GSEA on DE transcripts revealed that PMS CM induced upregulation of oxidative stress, cytokine signaling, lipid metabolism, IFN pathways, and senescence-related transcripts in Ctrl iNSCs. The effect of the PMS iNSC CM was abrogated in PMS iNSCs treated with ABT-263 (Figures 6C–6E). Additionally, PMS CM suppressed cell-cycle-associated transcripts in Ctrl iNSCs, which was reversed in ABT-263 CM (Figures 6C and 6D).

PMS iNSCs have a functional inflammatory and senescent phenotype that propagates inflammation and senescence to neighboring cells. Importantly, ABT-263 treatment did not eradicate the senescent cell cluster outright but substantially decreased the expression of senescence, inflammatory, and IFN signaling transcripts. This highlights the potential of ABT-263 as a senomorphic agent capable of mitigating neuroinflammation and cellular dysfunction in PMS models.

### Spatial enrichment of DARGs in lesion-associated niches of the human MS brain

**Figure 7.**
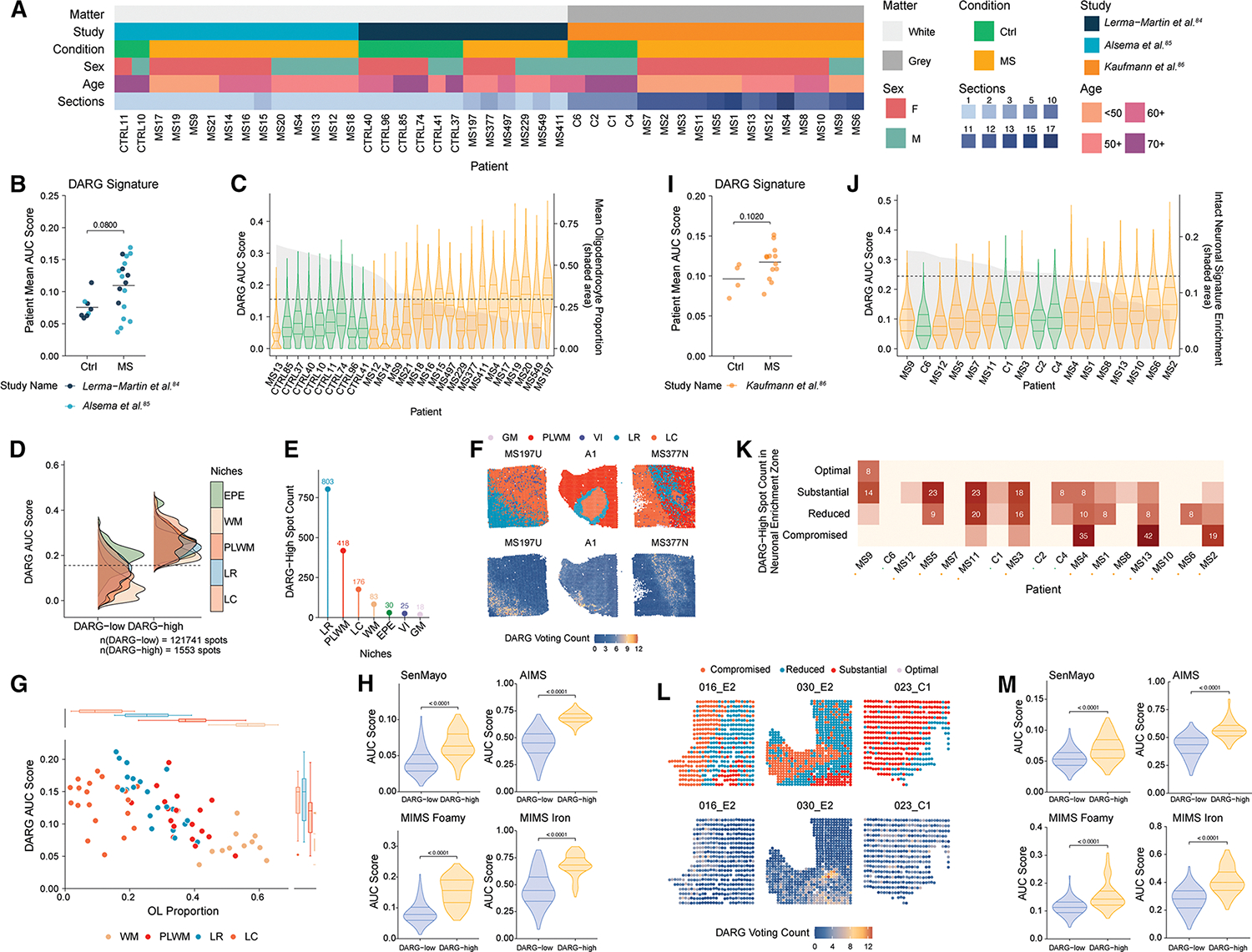
Spatial transcriptomics reveals consistent DARG enrichment in lesion-associated regions (A) Overview of re-analyzed patient cohorts. (B and I) Comparison of patient-level mean DARG signature enrichment between Ctrl and MS patients across contributing white (B) and gray (I) matter samples. Significance was assessed by the two-tailed Wilcoxon signed-rank test. (C and J) Intra-patient distribution of spot-level DARG signature enrichment. Samples are decreasingly ordered by their mean oligodendrocyte content (C) or neuronal enrichment (J). The horizontal dashed line indicates the data-driven confidence limit on AUC background noise. (D) Difference in DARG signature enrichment between DARG-low and DARG-high spots across niches in WM samples. (E) Number of DARG-high spots across niches in WM samples. (F) Representative WM sections, delineating spatial niches and per-spot presence of DARG signature. (G) Relationship between oligodendrocyte proportion and DARG signature enrichment. A dot represents a niche’s mean value per patient. (H and M) Comparison of signature enrichments for SenMayo^74^ and lesion-associated glia (AIMS and MIMS^16^) between DARG-low and DARG-high spots in white (H) and gray (M) matter samples. Significance was assessed by an unpaired two-tailed *t* test. (K) Number of DARG-high spots across neuronal enrichment zones in GM samples. (L) Representative GM sections, delineating neuronal enrichment zones and per-spot presence of DARG signature.

To characterize the *in situ* distribution and spatial localization of DARGs in MS, we re-analyzed three publicly available spatial transcriptomic datasets derived from post-mortem human brain tissue. Two focusing on subcortical white matter (WM)^84,85^ and one on cortical GM,^86^ collectively encompassing over 200 tissue sections from 31 MS patients and 12 controls (Figure 7A; Table S7).

In WM samples from Lerma-Martin et al.^84^ and Alsema et al.,^85^ we observed a consistent elevation of DARG area under the curve (AUC) scores across the MS tissues at the patient level (Figure 7B), with variability among individuals reflecting differences in disease stage and sampling location. At the spot level, DARG scores were higher in MS relative to controls, a pattern robust across datasets (Figures S7A–S7C). Higher DARG scores were inversely correlated with oligodendrocyte proportions (Figure 7C).

We identified 1,553 DARG-high spots (1.26%) across WM sections, with enrichment increasing from the lesion core (LC) to the lesion rim (LR) and perilesional WM (PLWM) (Figures 7D–7F, S7D, and S7E). We also identified DARG-high spots localized to the ependymal niche, suggesting a role in lesion formation.^87^ Within individual patients, DARG scores progressively increased from PLWM to LC, concurrent with decreasing oligodendrocyte proportions and rising densities of astrocytes and microglia (Figures 7G and S7F–S7I).

Further examination revealed significant enrichment of “astrocytes inflamed in MS” (AIMS) and “microglia inflamed in MS” (MIMS) signatures^16^—including the foamy and iron-associated phenotypes—within DARG-high spots (Figure 7H). These regions also exhibited elevated expression of RG-like and senescence-associated transcripts, as defined by the SenMayo panel,^74^ supporting the existence of a senescent, multicellular niche associated with chronic inflammation.^16^

We further analyzed the cortical GM dataset from Kaufmann et al.,^86^ identifying 321 DARG-high spots (0.39%) across GM sections. We recapitulated similar patterns. DARG AUC scores were elevated in MS compared with controls (Figure 7H), and higher DARG enrichment correlated with reduced tissue integrity as measured by a GM-specific neurodegeneration score (Figure 7I). DARG-high spots localized predominantly to lesion boundaries, with the extent of DARG-positive areas increasing alongside neurodegenerative burden (Figures 7J, 7K, and S7J–S7P). Enrichment of senescence, AIMS, and MIMS signatures in DARG-high spots (Figure 7L) underscores a conserved presence of these cells across cortical and subcortical regions. Taken together, the re-analysis of this large GM dataset recapitulated key features observed in WM, further strengthening the evidence for the presence of DARGs in the human MS brain.

Our findings reveal that DARGs constitute a discrete subset of disease-associated cells predominantly localized to lesion niches in the MS brain. Their spatial enrichment—alongside glial activation and tissue damage—suggests a potential role in sustaining chronic inflammation.

### Integrative multi-omics reveal regulons defining inflammatory RG-like cells in PMS iNSCs

To explore the epigenetic mechanisms behind the transcriptomic signature of the PMS cluster 5, we integrated the RNA-seq data with single-nucleus chromatin accessibility data (snATAC-seq). For a high proportion of cells, the matched RNA and ATAC quantification were distributed proportionally across clusters (Figure S8A). Using selected data-driven parameters, we identified 8 stable ATAC-seq clusters (Figures S8B–S8D).

**Figure 8.**
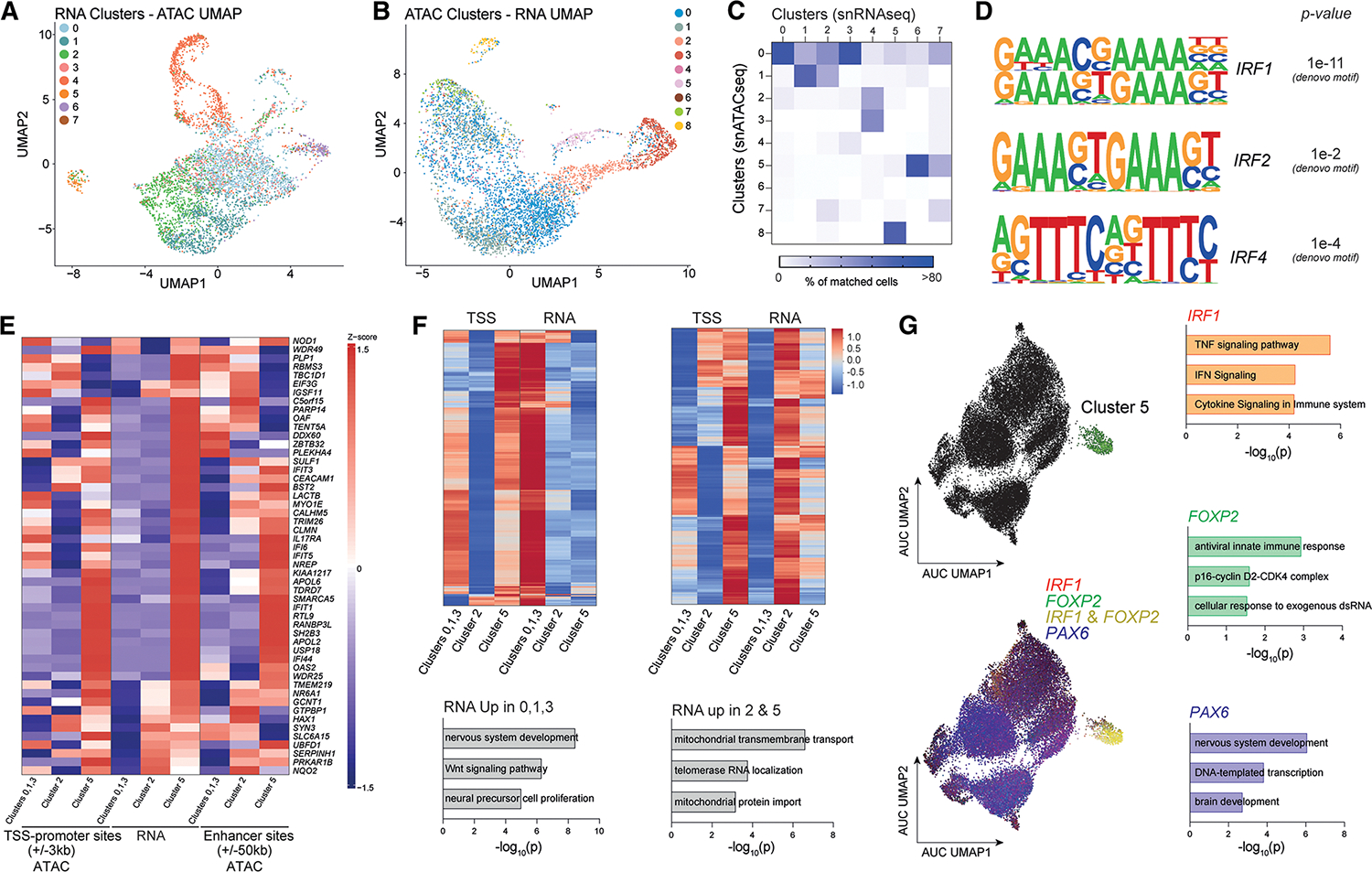
Multi-omics RNA/ATAC integration with further epigenetic characterization of cluster 5 cells (A) ATAC UMAP of the overlaid localization of RNA-seq clusters. (B) RNA UMAP of the overlaid localization of snATAC-seq clusters. (C) Heatmap of the percentage of matched assignations of cells across the snRNA-seq and snATAC-seq clusters. (D) Enriched motifs of signature and differentially accessible genes identified on RNA cluster 5 and ATAC cluster 8. (E) Heatmap of ATAC and RNA of *IRF1* targets identified as DE between grouped RNA clusters 0, 1, and 3 vs. cluster 2 and cluster 5. (F) Heatmap and GSEA of marker genes between clusters 0, 1, and 3 vs. 2 and 5. (G) SCENIC GRN inference summary and selection of IRF1-specific regulons, corroborated with enrichment analysis of regulons.

We projected the RNA clusters onto the ATAC UMAP and the ATAC clusters onto the RNA UMAP to evaluate modality concordance, highlighting the agreement between RNA cluster 5 and ATAC cluster 8 (Figures 8A–8C). We confirmed that the inflammatory ATAC cluster 8 was primarily composed of PMS cells (Figure S8E).

Differentially accessible regions (DARs) specific to inflammatory ATAC cluster 8 were associated with immune processes, IFN signaling, and cytokine production (*IRF3* and *STAT2*), and DARs that lost accessibility were associated with genes pertaining to neuron and astrocyte differentiation and neural crest cell fate specification (*SOX4* and *SOX8*) (Figure S8F; Table S8).

We identified significantly enriched common TFs, including *p53*, *E2A*, and *SMAD2*, in both PMS fibroblasts and iNSCs from the WGBS and from the predicted signature genes of RNA cluster 5 (Figure S8G). As *p53* and *E2A* are implicated in promoting immune function, senescence, and in mediating IFN responses,^88,89^ our findings suggest that the chromatin accessibility for RNA cluster 5 closely predicts its RNA expression signature.

Since IFN-associated TFs were prominent in RNA cluster 5 and ATAC cluster 8 (Figure 8D), we next investigated the chromatin accessibility at the promoter regions. We identified increased accessibility of *IFIT1* in ATAC cluster 8, corresponding to increased expression in RNA cluster 5 (Figure S8H). *IRF1* is a key TF implicated in facilitating TNF-α-induced senescence and is known to be anti-proliferative and pro-inflammatory.^90^ Within IRF1 targets, we found clear associations between RNA expression (RNA cluster 5) and chromatin accessibility near TSS (±3 kb), as well as in potential regulatory regions (±50 kb from TSS) in ATAC cluster 8 (Figure 8E). These data support the hypothesis that cells in RNA cluster 5 have permissive chromatin that underlies persistent activation of IFN responses via IRF1.

The pseudotime analysis predicted RNA cluster 2 being closely related and strongly interacting with RNA cluster 5. We next examined if a similar gene expression and chromatin accessibility signature could be identified in both clusters.

Cluster 2 showed overlapping inflammatory genes (*HAX1* and *SERPINH1*) with similar chromatin accessibility changes to cluster 5 (Figure 8E). GSEA on shared ATAC signatures suggested that common gains of accessible regions were associated with stress response and blood-brain barrier (BBB) maintenance, whereas loss of accessible regions correlated with Notch and bone morphogenetic protein (BMP) signaling and senescence pertaining to cell proliferation (Figures S8I and S8J). Not all changes in chromatin accessibility correlated directly to variation in mRNA (Figure S8J). To validate our ATAC findings, we generated independent snATAC-seq on additional samples (Table S1). The new snATAC-seq dataset revealed the presence of 9 clusters, with clusters 1, 2, and 4 being significantly enriched in PMS cells and cluster 8 being enriched with control cells (Figure S8K). We found associations between chromatin accessibility (±50 kb) in ATAC clusters 1, 2, and 4 (6,313 cells in Ctrl vs. 10,945 in PMS) along with the P3 inflammatory RNA cluster 8 from the scRNA-seq analysis in *IRF1* targets (Figure S8L). We identified increased IRF1 target expression in small (723 cells in Ctrl vs. 18 in PMS) control-enriched cluster 8, which overlapped with the C3 inflammatory cluster 12 (Figure S8L). This further confirms the existence of an epigenetically dysregulated IFN response in cells from people with PMS.

We subdivided gene sets based on RNA upregulation in RNA clusters 0, 1, and 3 and depletion in RNA clusters 2 and 5, finding enrichment for Wnt signaling and NSC maintenance (Figure 8F). Coordinated upregulation in RNA clusters 2 and 5 showed enrichment in terms associated with mitochondrial transmembrane transport, protein import, and telomerase RNA localization (Figure 8F), supporting a close interaction between RNA clusters 2 and 5, with cluster 5 being specifically IFN-responsive.

Lastly, we inferred single-cell regulatory networks using single-cell regulatory network inference and clustering (SCENIC).^91^ We identified RNA cluster 5 as defined by *IRF1* and *FOXP2 regulons* that were associated with genes regulating TNF and IFN signaling, as well as the p16-cyclin complex related to senescence (Figure 8G; Table S8). *PAX6*, a major regulon across all iNSC clusters, was associated with nervous system development, supporting their RG-like state. Overall, RNA cluster 5 is consistently defined by gene regulatory patterns that are associated with an IFN-responsive and senescence state.

In summary, our integrative multi-omics analysis uncovers a distinct, epigenetically dysregulated, IFN-responsive, and senescent RG-like cell population in PMS regulated by *IRF1*. PMS iNSCs propagate their inflammatory and senescent phenotype through the SASP, which can be effectively inhibited by senolytic treatment. Integration with publicly available datasets uncovered DARGs significantly enriched in chronic active lesion areas. These findings highlight a novel cellular axis that may drive smoldering inflammation in PMS.

## DISCUSSION

PMS is a complex neuroinflammatory and neurodegenerative disorder resulting from the interaction between environmental factors and genetic predisposition. MS severity has been connected to variants involved in mitochondrial function, synaptic plasticity, and cellular senescence in CNS-expressed genes.^19^ To investigate how intrinsic glial cell dysfunction in PMS contributes to disease pathology, we generated directly reprogrammed iNSCs.

Our characterization revealed that PMS-derived iNSCs recapitulate key features of the disease, including elevated inflammatory signaling and senescence gene expression, aligning with prior reports.^17,18^ iNSCs preserve shorter telomeres, indicating retention of aging-related features, and maintain DNAm age—suggesting a persistent epigenetic memory of disease.

Epigenetic profiling showed global hypomethylation in PMS cells, particularly at promoters regulated by inflammatory TFs, including STAT6 and NF-κB, mirroring patterns observed in aging tissues.^92^ These epigenetic changes, associated with aging and genome instability,^92^ were also found in PMS fibroblasts, indicating persistent, disease-associated epigenetic dysregulation. Similarly, recent work has identified the existence of a disease-associated epigenetically altered pro-inflammatory memory phenotype in human astrocytes found in chronic MS lesions.^93^

Concordantly, hypomethylated regions in PMS cells were enriched for immune response pathways, consistent with prior work identifying immune-related epigenetic signatures in MS blood and CNS tissue.^94^ We also identified hypomethylation in genes governing lipid metabolism, which have been implicated in PMS phenotypes.^18,94^ Our findings demonstrated that disease-associated epigenetic changes originate in fibroblasts and persist in iNSCs. Interestingly, previous work has suggested signatures of cell stress in fibroblasts from people with MS, which may alter their epigenetic profile,^95^ suggesting human iNSC methodologies^48^ provide an excellent platform to study disease-associated epigenetic alterations.

The relationship between senescence and IFN signaling is well established.^96^ IFN responses can be triggered by various stimuli, including cytosolic double-stranded RNA/DNA from stress, apoptosis, or microbes, activating pro-inflammatory pathways.^97,98^ In aging brains and neurodegenerative diseases, aberrant IFN activation promotes inflammation and immune cell infiltration, leading to neurodegeneration.^99–102^ Our findings of hypomethylation and increased chromatin accessibility at relevant regulatory elements in PMS fibroblasts and iNSCs support the idea of an inherent predisposition toward an exaggerated IFN response. Hypomethylation is associated with increased potential for gene transcription and has been observed in a variety of aged tissues.^92^

From the sc data, we pinpoint an RG-like subset capable of amplifying inflammation via secretion of paracrine factors. Despite being a small fraction, RNA cluster 5 may induce paracrine senescence and inflammation via secreted factors, as seen in mouse studies where small amounts of senescent cells can have profound implications in neurodegenerative-like disease.^103^ Our work demonstrates the capability of PMS iNSC CM to promote upregulation of senescence and inflammatory-associated genes in Ctrl iNSCs. Ligand-receptor predictions also suggest cluster 5 induces inflammatory-associated signatures and inhibits NSC maintenance in the other iNSC clusters. Supportively, our prior work shows PMS iNSC CM can induce neurite retraction and neuronal apoptosis, highlighting the pathological relevance of secreted inflammatory factors.^18^

The basis of the RG-intrinsic dysfunctional phenotype in PMS remains unclear, but epigenetic signatures are evident in donor fibroblasts. Neurodegenerative diseases are strongly linked to viral exposure, especially Epstein-Barr virus (EBV), which increases MS risk.^104,105^ Viral exposure and chronic inflammation may induce widespread epigenetic changes in cell types, including fibroblasts, which are stressed in MS.^95^ Human endogenous retroviruses (HERVs), activated by inflammation, trigger IFN responses and are associated with neurodegenerative diseases and brain injuries.^106,107^ We identified epigenetic sites with increased accessibility related to IFN response, cytokine production, and stress markers in RNA cluster 5 (ATAC cluster 8), mainly composed of PMS cells. This remodeling also involved increased accessibility at p53 binding sites, linked to cellular stress and sustained IFN signaling.^88^

Using MS post-mortem brain datasets, we assessed the expression of RG genes and identified non-neurogenic RG-like cells within astrocyte clusters. Recent work has demonstrated that astrocytes exhibit plasticity in injury situations, de-differentiating into replicating NSC/RG-like cells in human lesions with BBB rupture.^43^ Furthermore, this has been previously validated in rodent models where epithelial injury allows for neural precursors to de-differentiate into multipotent NSCs in the olfactory epithelium.^108^ Based on these studies, astrocytes in PMS may be undergoing (1) a de-differentiation (or de-maturation) process, where they begin to express cell cycle and early RG-like cell markers due to exposure to chronic inflammation, and (2) a resurgence as non-neurogenic RG-like cells at the level of disease-associated, ectopic, non-canonical niches.

DARGs were confirmed in post-mortem MS tissue, especially within chronic active lesions, where they display IFN and senescence gene signatures. Spatial transcriptomics showed that DARGs are enriched in LRs, PLWM, and even the ependymal niche, suggesting their potential involvement in lesion formation and progression. This non-neurogenic RG-like population correlates with more aggressive, slowly expanding lesions featuring the hallmarks of chronic demyelination, remyelination failure, and axonal loss.^109,110^

Our study reveals epigenetic changes in somatic fibroblasts isolated from people with PMS, retained after direct reprogramming into iNSCs. These epigenetic alterations are associated with de-repression (hypomethylation and increased chromatin accessibility) of IFN signaling and response as well as inflammation. Importantly, we demonstrate that this inflammatory and senescent phenotype propagated by DARGs can be mitigated through senolytic strategies.

In conclusion, our integrated *in vitro* and tissue analyses reveal DARGs, which display hallmark features of inflammation and senescence. Their enrichment in chronic lesions and association with ongoing neurodegeneration suggest that targeting this cellular axis may provide new avenues for therapeutic intervention aimed at disrupting disease progression in PMS.

### Limitations of the study

While we successfully generated cell lines from individuals with PMS, variations in genetic backgrounds, sex, and age among these lines represent confounding factors, inducing a limitation of our study. Future investigations involving patient-derived cells from larger and more diverse cohorts are necessary to validate and generalize our findings. Additionally, although our analyses encompassed both patient and control cell lines, further validation in biological tissue is required. This includes a comprehensive assessment of protein markers for DARGs in the PMS brain. Lastly, further functional studies in novel human and animal models of MS-like disease or lesions are necessary for confirming the relevance and mechanism of DARGs *in vivo*.

### RESOURCE AVAILABILITY

#### Lead contact

Further information and requests for resources and reagents should be directed to and will be fulfilled by the lead contact, Prof. Stefano Pluchino (spp24@cam.ac.uk).

#### Materials availability

This study did not generate new, unique reagents.

#### Data and code availability

- All original code has been deposited at Zenodo and is publicly available at DOI https://doi.org/10.5281/zenodo.15581507 and DOI https://doi.org/10.5281/zenodo.15584699.
- All data generated for this study, in raw and processed format, are publicly available on the Gene Expression Omnibus (GEO) under accessions GEO: GSE297192 (bulk RNA-seq), GEO: GSE251839 (WGBS), GEO: GSE297365 (scRNA), and GEO: GSE297690 (scATAC).
- Further data mining of processed data may be performed on https://mohorianulab.org/shiny/pluchino/DARG_PMS/ and https://genomicspark.shinyapps.io/shinyApp/.
- Any additional information required to reanalyze the data reported in this paper is available from the lead contact upon request.

## STAR★METHODS

Detailed methods are provided in the online version of this paper and include the following:

### EXPERIMENTAL MODEL AND STUDY PARTICIPANT DETAILS

#### Human cells

The cohort consists of 3 PMS and 3 healthy controls between 25 and 63 years of age. The cohort includes representation from both genders, distributed across PMS and control groups (Table S1). We were unable to investigate the potential influence or association of sex on the findings due to the small donor size. PMS fibroblasts were provided by the New York Stem Cell Foundation (NYSCF) Research Institute through their Repository (http://www.nyscf.org/repository).^129^ Patients were recruited at the Tisch Multiple Sclerosis Research Centre of New York, upon informed consent and institutional review board approval (BRANY). PMS donors underwent clinical assessment when recruited for the study. Control fibroblasts C1 and C2 (Table S1) were generated from adult dermal fibroblasts after obtaining consent and ethical clearance by the ethics committee of the University of Würzburg, Germany.

#### Generation and culturing of induced neural stem cells

iNSC lines were generated and quality controlled as described in Ionescu et al.^18^ and Meyer et al.^48^ iNSCs were maintained in neural induction media (NIM) consisting of DMEM/F12 and Neurobasal (1:1) (Thermo Fisher), supplemented with N2 supplement (1x) (Thermo Fisher), 1% GlutaMAX (Thermo Fisher), B27 supplement (1x) (Thermo Fisher), CHIR99021 (3 μM) (Cell Guidance Systems), SB-431542 (2 μM) (Cayman Chemical), and hLIF (10 ng/ml) (PeproTech) until 70% confluent, then lifted using accutase, spun at 300 × g for 3 mins, and plated onto growth factor reduced (GFR) matrigel matrix coated plates (Corning) (1:20 in DMEM/F12) with Y-27632 (10 μM) (Miltenyi Biotec) between 1:3–1:5 in NIM media. Media was changed every second day as needed. Experiments were performed on cells from passages 15–35.

#### Fibroblast maintenance

Fibroblasts were maintained in growth medium comprised of DMEM GlutaMAX (Thermo Fisher) supplemented with 10% fetal bovine serum, 1% non-essential amino acids and 1 mM sodium pyruvate (Thermo Fisher) at 37°C with 5% CO_2_ and fed every 3–4 days. After reaching 90% confluency the fibroblasts were detached with trypsin-EDTA 0.05% for 5 min followed by neutralization in DMEM and spun down at 300×g for 5 min. They were split 1:4 into growth media onto tissue-culture treated plasticware.

### METHOD DETAILS

#### Immunocytochemistry of iNSCs

Cells were stained as described in Ionescu et al.^18^ Cells were plated at a density of 50,000 cells/cm^2^ on GFR-matrigel-coated glass coverslips. Cells were fixed for 10 minutes with 4% paraformaldehyde (Thermo Fisher) then permeabilized for 10 minutes in PBS with 0.25% Triton-X100 (Thermo Fisher). Cells were then blocked for 1 hour using 1% normal donkey serum or goat serum (Thermo Fisher) in PBS with 0.1% Triton-X100. Cells were incubated with the following primary antibodies in the above blocking buffer:

Nestin (Novus Biolgicals, 1:500), SOX2 (Abcam, 1:500), OCT4 (Santa Cruz, 1:200), PAX6 (BioLegend, 1:100), Vimentin (antibodies.com, 1:1000), and KI67 (Abcam, 1:250) were applied in PBS with 0.1% Triton-X100 at 4°C overnight. Coverslips were washed three times for 10 minutes each in PBS with 0.1% Triton-X100 then incubated with species-appropriate secondary antibodies (1:1000) in blocking buffer for one hour at room temperature. Coverslips were washed again as above then stained with DAPI in PBS (300 nM, Thermo Fisher) for five minutes then washed in PBS. Coverslips were mounted onto glass slides using Fluoromount-G (Thermo Fisher). Images were taken on a Leica DMI400B microscope at 40X in oil immersion. Ten region of interest images were taken from two individual coverslips from each line for quantification.

#### RNA isolation and RT-PCR

Total RNA from all cell types was isolated using the RNeasy Mini Kit (Qiagen) following manufacturer’s instructions. iNSC quality control was performed using PCR analysis described in Ionescu et al.^18^ 300 ng of total RNA was reverse-transcribed using the High-Capacity cDNA Reverse Transcription Kit (Thermo Fisher) according to the manufacturer’s instructions using a 20 μL reaction volume on a T100 Thermal Cycler (BioRad). The RT-PCR reaction was made using DreamTaq buffer (Thermo Fisher), dNTPs (2 mM each, Thermo Fisher), forward and reverse primers (0.5 μM), DreamTaq Hot Start DNA Polymerase (Thermo Fisher), and finished to 24 μL with water. 1 μL of cDNA from each sample was loaded into the PCR reaction. *OCT4* reactions were cycled at: 95°C for 3 mins; x30 cycles of 95°C 30 sec, 60°C 30 sec, 72°C 1 min; 72°C 5 min, hold at 4°C. *SeV*, *KOS*, *KLF4*, *SOX2*, *PAX6*, and *NES* reactions were cycled at: 95°C for 3 mins; x30 cycles of 95°C 30 sec, 55°C 30 sec, 72°C 1 min; 72°C 5 min, hold at 4°C. Samples were diluted with Gel Loading Dye (New England BioLabs), and 5 μL was loaded into a 2% agarose gel (Thermo Fisher) in 1X TAE buffer (MP Biomedicals). TrackIt 100 bp DNA Ladder (Thermo Fisher) was used to assess band size. DNA was visualized using Gelred Stain (Biotium) in the gel and imaged on a BioRad XR GelDoc Imager.

#### mRNA sequencing, analysis, and inference of gene regulatory networks (GRNs)

iNSC lines, between passages 15–30, were plated at 250,000 cells per well in GFR-coated 12-well plates. After 24 hours, the media was refreshed with new NIM. Cells were harvested in RLT lysis buffer 72 hours after plating then frozen at −80°C until extraction.

For the senolytic conditioned media experiments PMS iNSCs were cultured at 500,000 cells per well in GFR-coated 6-well plates. After 24 hours, the media was refreshed with new NIM either with vehicle control DMSO or 3 μM of ABT-263 (Selleck Chemicals). After 48 hours the media was removed and refreshed with fresh NIM. Cells were cultured for an additional 48 hours, and CM was collected, spun at 300 × g for 5 mins to remove cellular debris, and stored at −80°C until use. iNSCs were collected in RIPA for immunoblotting. Control iNSCs were plated into GFR-coated 12-well plates at 250,000 cells/well. 24 hours after plating, the media was replaced with 750 μL of PMS or PMS+ABT-263 CM. Cells were harvested in RLT lysis buffer after 48 hours of CM treatment and then frozen at −80°C until extraction.

RNA extraction was performed according to standard steps described for the RNeasy kit, followed by DNase treatment (Qiagen). RNA was quantified using the NanoDrop 2000c instrument. Illumina Sequencing libraries were prepared using the TruSeq low sample protocol from 1 μg of total RNA (Illumina, San Diego, CA, USA). The resulting libraries were sequenced in paired-end mode, on 150 nts reads on an Illumina NovaSeq X instrument.

The quality checking of the samples was assessed using fastQC v0.12.11, applied on raw files; the outputs were summarized using multiQC 1.14.^112^ Initial sequencing depths ranged from 30M to 44M reads; subsampling without replacement, done using seqtk 1.3-r106,^130^ was performed to 20M reads, to avoid inconsistencies caused by uneven sequencing depths.^131^ All samples were aligned to the GRCh38.p13 genome using STAR 2.7.10a (paired-end mode).^115^ Expression quantification was performed using featureCounts v.1.6.3.^116^ The distribution of signal across transcripts was assessed on the UCSC genome browser. The tracks (bigwig format) were built from the bam files using samtools 1.17.^132^ Next, noisyR 1.0.0^113^ was used to estimate and remove noise from the count matrix; the raw expression levels were normalized using quantile normalisation.^133^ DEGs were identified using edgeR^117^ and DESeq2^118^; due to the noise correction the DEG calling converged; p-values were adjusted using Benjamini-Hochberg multiple testing correction. bulkAnalyseR 1.1.0^50^ was used to build a shareable interface for the analysis and visualization of data.

The gene regulatory networks (GRNs) were predicted, and their dynamics assessed, on the bulk RNA-seq data using a bulkAnalyseR ShinyApp.^50^ Additional analyses were performed using GENIE3^119^ and visNetwork^120^ to visualize subgraphs according to selected pathways and a maximum of 30 edges. To assess the global trend in co-variation of expression, for genes annotated to the selected pathways, density plots were created, per pathway, on the weights of the edges in the larger GRNs (corresponding values in the global adjacency matrix).

#### Immunoblotting

iNSCs were homogenized in 10X RIPA buffer (Abcam) supplemented with 100X protease and phosphatase inhibitors (Thermo Fisher). Protein concentration was assessed using a BCA assay (Thermo Fisher). Equal protein amounts (25 μg) were resolved by SDS-PAGE on Bolt^™^ Bis-Tris Plus pre-cast 4–12% gradient gels (Invitrogen) and transferred to 0.45 mM polyvinylidene fluoride (PVDF) membranes (Thermo Fisher). Membranes were blocked with TBS blocking buffer (LI-COR Biosciences) and immunoblotted with the indicated antibodies: mouse anti-p16^Ink4a^ (Invitrogen) at 1:500, rabbit anti-p21 (Thermo Fisher) at 1:500, rabbit anti-GDF15 (Proteintech) at 1:1000, and mouse anti-b-actin (Sigma) at 1:5000 in TBS blocking buffer (LI-COR Biosciences) with 0.1% Tween, followed by fluorescent secondary antibodies IRDye 680RD Goat anti-Rabbit or IRDye 800CW Goat anti-Mouse (LI-COR Biosciences) at 1:10,000 in TBS blocking buffer (LI-COR Biosciences) with 0.1% Tween and 0.01% SDS. The immunoblots were visualized with the ChemiDoc MP Imaging system (Bio-Rad). Densitometric analysis was conducted with Fiji by ImageJ. Protein targets were normalized to β-actin.

#### SPiDER-β-gal measurements

Cells were plated on black-walled, clear bottom 96-well plates (Thermo Fisher) at 15,000 cells/well and maintained in culture for 5 days. Expression of senescence-associated β-galactosidase was measured by a SPiDER-β-gal-based cellular senescence plate assay kit (Dojindo) according to manufacturer’s instructions. Briefly, cells were washed with PBS, stained with 1 μg/mL Hoechst (Sigma-Aldrich) to measure cell number, and washed again before the fluorescence intensity was measured at 358 nm Ex / 461 nm Em. Cells were then lysed with the provided buffer and the SPiDER-β-gal stain was added and incubated at 37°C overnight. Fluorescence intensity was measured at 520ex/565em. The SPiDER-β-gal fluorescence intensity of each well was corrected for the autofluorescence of empty wells and normalized to the Hoechst fluorescence intensity of the respective well to normalize for cell number. The resulting average SPiDER-β-gal/Hoechst fluorescence intensity of each cell line was normalized to that of healthy control cell lines.

SPiDER-β-gal immunofluorescence was performed based on the manufacturer’s instructions (Dojindo). Before seeding, control and PMS iNSCs were treated with DMSO or 3 μM ABT-263 (Selleck Chemicals) for 48 hours. iNSCs were next cultured on GFR-coated 24-well coverslips at 80,000 cells/well. After 24 hours cells were washed with HBSS, then given the SPiDER-β-gal working solution in NIM media for 30 minutes at 37°C in a 5% CO_2_ incubator. The working solution was removed, cells were washed with PBS, then fixed with 4% PFA for 10 mins. Coverslips were then stained with DAPI in PBS (300 nM, Thermo Fisher) for five minutes and washed in PBS. Coverslips were mounted onto glass slides using Fluoromount-G (Thermo Fisher). Images were taken on a Leica DMI400B microscope at 40X in oil immersion. Quantification was performed using 4 regions of interest (ROIs) and batch analysed using CellProfiler.

#### EdU Immunofluorescence

EdU staining was performed based on the manufacturer’s instructions (Thermo Fisher). Before seeding, control and PMS iNSCs were treated with DMSO or 3 μM ABT-263 (Selleck Chemicals) for 48 hours. iNSCs were next cultured on GFR-coated 24-well coverslips at 60,000 cells/well. After 24 hours iNSCs were treated with 10 μМ EdU for two hours then fixed using 4% PFA for 10 mins. Cells were washed with 3% BSA in PBS and then permeabilized for 20 minutes with 0.5% triton in PBS. Coverslips were incubated with the Click-iT EdU staining solution for 30 mins, washed again with 3% BSA in PBS, incubated with DAPI in PBS (300 nM, Thermo Fisher) for 5 minutes, washed in PBS, then mounted onto glass slides using Fluoromount-G. Images were taken on a Leica DMI400B microscope at 20X in oil immersion. Quantification was performed using 4 ROIs and batch analysed using CellProfiler object count function.

#### Cell cycle analysis

iNSCs were plated at a density of 80,000 cells/cm^2^ on GFR-coated plates. After 3 days cells were lifted using accutase and then pelleted at 500 × g for 5 minutes. The cells were fixed in 70% ethanol for 30 minutes on ice and then pelleted at 850 × g for 5 minutes. The cell pellet was resuspended in RNase (100 ug/mL) for 15 minutes and incubated at room temperature. Propidium iodide (1 ug/mL) was added to each sample and cells were analysed on a BD LSRFortessa with the flow rate on slow. 20,000 events were collected for each sample. The data were analyzed using FlowJo 10.9 software using the Dean-Jett-Fox approach.

#### Telomere length analysis

Relative telomere length was assessed using the Joglekar et al. protocol using quantitative PCR (qPCR) and comparison to that of a single copy gene.^134^ iNSCs were plated at a density of 80,000 cells/cm^2^ on GFR-coated plates. After 3 days cells were lifted using accutase and then pelleted at 500 × g for 5 minutes. DNA was isolated according to the DNeasy Blood & Tissue Kit (Qiagen) and quantified using the Nanodrop 2000c instrument. For initial optimization of the qPCR reaction, the DNA was diluted to three different concentrations (100 ng/μL, 25 ng/μL, 6.25 ng/μL), and it was determined that 100 ng/μL had the best efficiency for both the human β-globulin and telomere primers. Two PCR reactions were separately conducted, for human β-globulin the mastermix was made using 5 μL Fast SYBR Green (Thermo Fisher), 1 μL of hbg1 primer (3 μM), 1 μL of hbg2 primer (7 μM), and 2 μL of nuclease-free water. The reaction was cycled at 58°C annealing temperature along with a melt curve analysis using a QuantStudio 7 Flex (Thermo Fisher). For the telomere primers, the mastermix was made using 5 μL Fast SYBR Green (Thermo Fisher), 1 μL of telomere A primer (1 μM), 1 μL of telomere B primer (3 μM), and 2 μL of nuclease-free water. The reaction was cycled at 56°C annealing temperature along with a melt curve analysis using a QuantStudio 7 Flex (Thermo Fisher). Each sample was run in duplicate. Average telomere length was calculated as the ΔΔCT=(PMSaveragehbgCt–PMSaveragetelomereCt)–(ControlhbgCt–controlaveragetelomereCt).

#### Generation of human iPS-NSCs

Human iPSCs for line C3 (Table S1) were generated from human skin-derived fibroblasts using the CytoTune-iPS 2.0 Sendai Reprogramming Kit (Thermo Fisher) with hKOS (MOI: 5), hc-Myc (MOI: 5), hKlf4 (MOI: 3). iPSC colonies emerged between days 15–20 and were manually picked onto hESC-matrigel (Corning) coated wells. iPSC media was changed every day using mTeSR Plus media (STEMCELL Technologies). When confluent, cells were lifted using ReLeSR (STEMCELL Technologies) and split 1:10–1:20 onto hESC-matrigel coated plates in mTeSR Plus media. The P3 (Table S1) iPSCs were obtained from Douvaras et al.^111^

Human iPS-NSCs were differentiated following the protocol from Perriot et al.^135^ Briefly, iPSCs were plated at 70–80% confluence in a 6-well plate. Cells were scraped using a cell scraper into clumps and transferred into a well of a low-binding 6-well plate in neural induction media (DMEM/F12 + GlutaMAX [Thermo Fisher], 1X N2, 1X B27 without vitamin A [Thermo Fisher], 500 ng/mL of noggin [PeproTech], 20 μM SB431542, 4 ng/mL FGF-2 [PeproTech], and 2 μg/mL laminin [Sigma-Aldrich] with 10 μM of Y-2763) and incubated at 37°C for 6 hours until the generation of spheroids. After 6 hours the spheroid suspension was moved to 6-well plates coated with poly-L-ornithine (Sigma-Aldrich)/laminin (PO/L). Media was changed every other day and cells were further cultured for 10 days in neural induction media. Between 10–13 days numerous rosettes were visible in the culture. On day 10, media was changed to expansion media (DMEM/F12 + GlutaMAX, 1X N2, 1X B27 without vitamin A, 10 ng/mL FGF-2, 10 ng/mL EGF [PeproTech]). On day 13 rosettes were cut into squares using a needle and moved to PO/L coated plates with 10 μM Y27632. From here, cells are defined NSCs and are at passage 1. Expansion media was changed every other day and cells passaged every 6–7 days onto GFR-matrigel-coated flasks. For WGBS the iPS-NSC lines were collected at P5.

#### Whole-genome bisulfite sequencing (WGBS)

Genomic DNA was extracted from 100,000 fibroblasts, iNSCs, and iPS-NSCs using the DNeasy Blood and Tissue Kit (Qiagen). The quantity of DNA was measured using the Quant-iT PicoGreen method and victor X2 fluorometry (Thermo Fisher), and the integrity of the DNA was evaluated with Agilent genomic DNA screen tape. 500 ng of genomic DNA was used for sequencing. The sample quality control criteria for the WGBS library were set to have a DNA integrity number (DIN) score of 7.0 and above. The extracted DNA was fragmented to an average insertion size of 550 base pairs and the fragments were attached to end-repaired adapters. Genomic DNA was bisulfite converted using the EZ DNA methylation Gold kit (Zymo) following the manufacturer’s instructions. We next applied the xGen Methyl-Seq Lib Prep kit (Integrated DNA Technologies) to prepare the genomic DNA library. Library quality control was performed using qPCR (LightCycler 480) and TapeStation 4200 (D1000 screen tape).

The dataset comprises 8 samples (4 fibroblast lines and 4 iNSC lines), with sequencing depths varying from 213M to 408M, and an average of 315M reads per sample. Reads with adapter contamination were trimmed using Trim Galore (0.4.3)^136^ with options: –paired –q 25. Trimmed reads were aligned to the *H sapiens* reference genome (version hg38), using HISAT2^137^ (version built in the current stable version of Bismark 0.23.1^122^). A bisulfite-converted index (GA and CT conversion) was generated with default parameters. We identified 28M CpG sites per sample, with sequencing coverage varying from 24x to 39x, (an average of 30x coverage per sample). The bismark_methylation_extractor tool was used to summarize the methylation levels at CpG sites. After assessing the bias at 5’end regions using M-bias results, the first 2nts were excluded, as follows: bismark_methylation_extractor -p –ignore 2 –ignore_r2 –comprehensive –no overlap –bedGraph –counts –buffer_size 16G ($Aligned read bam file).

#### Identification of differentially methylated sites and regions

MethylKit^123^ was used for DMC and DMR quantifications, and fibroblast *vs* iNSC comparisons. A minimum threshold minimum of 10nts coverage for downstream DNA methylation analysis was set. The aligned reads were split into 100nt tiles (DMRs) using metilene.^124^ Differential methylation was calculated, applying a McCullagh and Nelder^138^ correction for overdispersion, as well as the sliding linear model (SLIM) proposed in methylKit to correct for multiple testing. Tiles with a q-value < 0.05 and over 20% methylation difference were called differentially methylated. We identified 4,743 genes hypomethylated only in PMS fibroblasts, 3,133 genes hypomethylated only in PMS iNSCs, with 2,344 hypomethylated genes shared between PMS fibroblasts and iNSCs. Analysis of hypermethylated genes with DMRs both located in the promoter regions and in proximity of TSS defined 2,946 hypermethylated genes in PMS fibroblasts (*vs* Ctrl), 858 hypermethylated genes in PMS iNSCs (*vs* Ctrl), and 291 shared hypermethylated genes (PMS fibroblasts *vs* Ctrl iNSCs). Motif enrichment analysis was performed using Homer (*findMotifsGenome.pl*).

Annotations relevant for the hg38 v6.4 of the *H sapiens* reference genome (genes, exons, introns, UTRs, and other annotations) were extracted using Homer annotation tools. AnnotatePeaks.pl DMR hg38^64^ was used to evaluate the distribution of methylation across the genome. Next, a comparative analysis of the DMRs/DMCs across tissue types, contrasting the control and PMS samples was performed. In addition to the number of methylated tiles per annotation category was calculated, as well as their distance to the closest Transcription Start Site (TSS). To calculate the epigenetic age, we applied the Shireby-Cortex,^52^ Hovarth,^53^ and Zhang,^54^ ageing clocks frameworks. For the Horvath and Zhang estimations Clockbase platform^139^ was used, relying on matched Illumina methyl array IDs. The DNA methylation levels (0–100%) per CpG probe, and the sample metadata were submitted to Clockbase, and the predicted clock age was downloaded as CSV format. For the Shireby-Cortex^52^ aging estimate, we downloaded the DNA methylation probes, and coefficient values and relied on matched Illumina methyl array IDs. We used total 347 DNA methylation CpG probes to predict epigenetic aging (> 20nts coverage).

#### Nuclei isolation, library preparation, and RNA sequencing

For the single-nucleus single-omics, (ATAC) and multiomics experiments, respectively, 2 ×10^6^ cells were harvested; nuclei were isolated following the manufacturer’s instructions with minor modifications. For senolytic experiments on lines C3 and P3 (Table S1) iNSCs were seeded at 1 million cells/well in a GFR-matrigel-coated 6-well plate. After 24 hours media was replaced with fresh NIM with either DMSO or 3 μM of ABT-263. After 48 hours the media was removed and replaced with fresh NIM, and after 24 hours the iNSCs were collected for sc-ATAC or mutliomics sequencing. Briefly, cells were lysed in 100 μL of freshly prepared lysis buffer (1 mM Tris-HCl [pH 7.4], 1 mM NaCl, 300 μM MgCl_2_, 0.01% Tween-20, 0.01% IGEPAL CA-630, 0.001% Digitonin, 0.1% BSA, 100 μM DTT, and 100 mU/μL RNase inhibitor) for 1 minute on ice, washed twice in 500 μL of wash buffer (1 mM Tris-HCl [pH 7.4], 1 mM NaCl, 300 μM MgCl_2_, 0.01% Tween-20, 0.1% BSA, 100 μM DTT, and 100 mU/μL RNase inhibitor), and the number of nuclei was assessed using the Countess II FL Automated Cell Counter (Thermo Fisher). For the sc-ATAC libraries, we removed the RNase inhibitor in both the lysis and wash buffers. Thereafter, approximately 16,000 nuclei were incubated with the transposase enzyme, loaded into Chromium Next GEM Chip H Single Cell Kit (10x Genomics). snATAC libraries were generated using Chromium Single Cell ATAC Reagent Kits User Guide v1.1 (10x Genomics) according to manufacturer’s instructions. For the multi-omics samples, following the transposase step, nuclei were loaded into Chromium Next GEM Chip J Single Cell Kit (10x Genomics), and then snATAC libraries were prepared using Chromium Next GEM Single Cell Multiome ATAC + Gene Expression Reagent Kits (10x Genomics) according to manufacturer’s instructions. The quality of the libraries was checked on the Agilent Bioanalyzer with High Sensitivity DNA kit (Agilent); per sample libraries were sequenced on Illumina Novaseq 6000 with target sequencing depths of 25,000 – 70,000 reads per nucleus.

For single-cell (sc)RNA-seq, cells were counted using a hemacytometer, 10,000 cells were loaded into Chromium Next GEM Chip G Single Cell Kit (10x Genomics), and scRNA libraries were generated with Chromium Single Cell 3’ Reagent Kits v3.1 (10x Genomics) according to manufacturer’s instructions. The quality of the libraries was checked on the 4200 Agilent Tapestation with High Sensitivity DNA kit (Agilent); per sample libraries were sequenced on Illumina Novaseq 6000 with target sequencing depths of 30,000 – 65,000 reads per cell.

#### Single-cell transcriptomics/epigenetics data pre-processing

Standard CellRanger pipeline (6.1.2) and CellRanger ARC (2.0.0) were applied for aligning reads to the aforementioned version of the *H sapiens* genome and for quantifying gene/peak expression. For the RNA component, intron-matching reads contributed to the gene expression levels. The processed gene expression matrix was imported in Seurat.^140^ Additional filtering was performed on the distributions summarizing the number of counts, features, and percentages of reads incident to mitochondrial and ribosomal genes, across cells, per sample (accepted cells satisfied the criteria: number of UMIs > 8,000, number of genes per cell > 1,000, log_10_ (genes per UMI) > 0.75). This retained cells with 15–40% RP ratios for the scRNAseq samples and 2–25% RP ratios for the snRNAseq data samples (Figure S3B). Across the dataset, a 20% MT ratio filter was applied (Figure S3C). A total of 26,138 cells across all samples passed filtering criteria with median UMI counts per cell of 22,296. The average number of cells per sample was 3,267 (range: 1,277 – 5,545), and the average number of genes per cell was 5,725 (range: 4,357 to 6,731) (Figures S3D and S3E).

Samples from lines C3 and P3 were filtered according to the following criteria: number of reads between 3000 and 60000; number of features greater than 1000; percentage of reads incident to ribosomal proteins between 15 and 40; percentage of reads incident to mitochondrial genes between 2 and 10. Post-filtering, on all retained cells, the MT and RP entries were excluded from the expression matrix, pre-normalization. The normalization of expression levels was based on log_2_ normalization (scale.factor = 10000). The cell cycle assignation was performed in Seurat using the ‘CellCycleScoring’ function and *a priori* defined gene set.

#### Clustering

Next, we applied the ClustAssess framework to determine optimal, data-driven parameters, starting with the number and type features according the stability of resulting partitions.^114^ We used Element-centric similarity,^114^ summarized on 30 iterations into Element centric consistency (ECC),^141^ to objectively assess stability.^140^ Highly variable features (N= 1,000 determined using the vst) approach, yielded optimal outputs. A 20-shared nearest neighbour (SNN) graph was constructed on the HGV PCA.^140^ To address batch effect across sc and sn quantifications, we applied Harmony.^121^ Clustering was performed using Louvain approach (resolution=0.2), implemented within Seurat^140^ v4.0.5. 8 clusters produced a stable partition on scRNAseq and snRNAseq components. We excluded the smallest cluster (133 driven by a specific sample (i.e. C1-specific cluster). A X2 test was used to assess the significance of proportions of cells for PMS *vs* control samples. Marker genes (on cluster *vs* complement and pairwise differential expression) were identified using the ‘findMarker’ function. The top 5 most positively differentially expressed genes were visualized in a heatmap.

Enrichment analyses were performed using gprofiler^125^ on markers called on a Wilcox test with a |log_2_(FC)| threshold of 0.25, an adjusted p-value (Benjamini Hochberg multiple testing correction) less than 0.05 and a minimum percentage of cells expressing the gene of 0.1, in either subset. The background set for the enrichment analysis comprised all genes expressed in at least 10 cells.

#### Voting schemes

A variable voting-scheme was used to identify cell subsets requiring a minimum number of expressed genes corroborated with a minimum average expression level. The voting scheme for radial glial gene signature includes cells expressing 6 out of 9 manually curated genes (Table S4) with a normalized expression threshold of 1; glial progenitor cells express 7 out of 10 genes (Table S4) with an expression threshold of 0.5; neural progenitor cells express 5 out of 7 genes (Table S4) with an expression threshold of 0.5. The voting scheme for IFN-α/β signaling is based on cells expressing 3 out of 6 genes (Table S4) with an expression threshold of 0.5; NOTCH1 signaling comprises cells expressing 13 out of 16 genes (Table S4) with no abundance threshold (i.e., presence/absence).

#### Re-analysis of *Schirmer et al. and Absinta et al.* single-nucleus RNA sequencing datasets

Raw fastQ files from the studies by Schirmer et al.^67^ and Absinta et al.^16^ were downloaded from ENA using fasterq-dump. The quality checking and mapping leading to the filtered feature-barcode matrices were performed as described above. For the Schirmer et al.^67^ dataset, cells with less than 4,000 features/genes were retained; an upper bound of 15,000 was employed for the maximum number of UMIs per cell; 5% is maximum proportion of fragments incident to mitochondrial DNA; 10% is the maximum proportion of reads incident to nuclear ribosomal genes. For the Absinta et al.^16^ dataset, cells with the number of features between 200 and 5000 were kept for subsequent steps of the analysis; an upper threshold of 20,000 UMI counts was used, and the maximum mt% was set to 5%.

Both datasets were normalized using SCTransform^142^; the Absinta et al.^16^ dataset was batch corrected using Harmony^121^ on the patient variable, with θ = 2. To detect stable partitions, on each separate dataset, ClustAssess^114^ was used with 20–50 iterations, assessing resolution parameters between 0.1 and 1.5 (0.1 increment steps). For the Schirmer et al.^67^ dataset, the top 4500 highly variable features yielded the most stable partitions; for the Absinta et al.^16^ dataset the top 3,500 highly variable features were selected. For both, the optimal resolution value was 0.6.

#### Pseudotime analysis

Monocle3^143^ was used to infer trajectories for the *in vitro* data, as well as for the single-cell data from the Schirmer et al.^67^ and Absinta et al.^16^ studies. To identify the start and the endpoint, a selection of genes was used in a voting approach. The manually curated set of genes, used for determining the starting region in the *in vitro* data, comprises *TOP2A, CENPF, UBE2C, ASPM, APOLD1* with an expression threshold of 2 and a tolerance of 1 gene, i.e. any one gene from the set maybe not expressed; for the ending region, genes *IFIT2* and *CDKN2A* were used, with an expression threshold of 1.5 and a tolerance of 0.

For the Absinta et al.^16^ and Schirmer et al.,^67^ larger subsets of genes were used; for the former, the following genes were used for the starting region: *LY6E*, *PPAN*, *FASN*, *CLU, SORD*, *TRAP1*, *TUBB2A*, *AP1S2*, *YBX3*, with an expression threshold of 0.5 and a tolerance of 4 genes; for the ending region, the following genes were used *ISG15*, *B4GALT5*, *IFITM3*, *SAR1A*, *KIAA1217*, *TRPC4*, *FGF4*, *B2M*, *ZC3HAV1*, *WARS*, *FN1*, *IFIT1*, with an expression threshold of 0.5 and a tolerance of 4 genes missing. For the Schirmer et al.^67^ dataset, the ending region was defined by genes *ISG15*, *B4GALT5*, *IFITM3*, *SAR1A*, *KIAA1217*, *TRPC4*, *FGF4*, *B2M*, *ZC3HAV1*, *WARS*, *DDX58*, with an expression threshold of 0.5 and a tolerance of 6 genes.

Using the ordering of cells based on their transcriptomic signatures, the ClustAssess stability framework was applied on gene expression levels. This yielded a stable number of gene-clusters, named gene modules, representing a precursor of GRN inference; the genes per module were further characterized from a pathway perspective (using gprofiler^125^), against GO terms, KEGG and REAC terms, and functional elements (TFs and miRNAs). Next, we chose three gene modules that characterized sections of interest on the trajectory-based UMAPs of the *in vitro* dataset. The genes within each module were used to create a proxy (a transcriptomic pattern) subsequently employed to identify homologue gene modules computed based on the *ex vivo* datasets, Schirmer et al.^67^ and Absinta et al.^16^, respectively. Briefly, we considered the percentage of genes present in the *ex vivo* gene modules using the three *in vitro* gene modules; to account for the variable number of genes for bo*th in vitro* and *ex vivo* modules the outputs are scaled by the size of the gene set, i.e. larger gene sets are penalized more than smaller gene sets. The pairwise comparison of gene modules relies on Fisher’s exact tests, using the *in vitro* data as baseline comparator. Benjamini-Hochberg (FDR) correction was applied to account for the multiple testing pyscenic^126^ was used to infer regulatory interactions, aligned with the metadata available for the *Homo sapiens* (hg38) reference genome. A docker container was used to generate a loom object from the existing Seurat object. Loompy was used^144^ to create a SCope object, explored using the SCope web application.

#### Cell-cell regulatory interactions and effects

NicheNet^76^ (v 2.0.4) was used to predict intercellular regulatory interactions, based on ligand-receptor databases (weighted_networks_nsga2r_final.rds). The correlative analysis, summarized as interaction scores, was applied on cluster-specific marker genes (differentially expressed genes). Further analyses were focused on cluster 5 (“inflammatory cluster”) assigned as sender cells *vs* receiver cells, as the remaining clusters, respectively. The analysis was performed separately on control and PMS iNSCs, respectively. The summary of interactions was visualized using circos plot (circlize library v.0.4.15).

#### Cytokine array

iNSCs were plated at a density of 100,000 cells/cm^2^ on GFR-coated plates. Media was collected on day 5 from each line. The Human Cytokine Antibody Array C5 (RayBiotech) was used for semi quantitative detection of 80 proteins according to manufacturer’s instructions. Overnight incubation was performed for steps when the option was given. Membranes were exposed using a Gel Doc XR imager (BioRad). Blots were analyzed using the Protein Array Analyzer macro for ImageJ (written by Gilles Carpentier, 2008). The relative quantity of each protein was normalized to the positive and negative controls included on the array. The array was performed once for each iNSC line. Control lines were averaged together to generate a fold change comparison over PMS iNSC lines. To visualize the results, we calculated the Z-score per cytokine using the heatmap function in an R environment.

#### snATACseq analysis

CellRanger ARC2.0.0 (multi-omics) and CellRanger ATAC2.0.0 (snATAC only) were used to map reads and quantify expression of peaks for the single-nuclei ATAC-seq datasets. The peak calling was performed on pseudobulked inputs, comprising cells with at least 100 reads sequencing depth. Union peaks (peaks called in at least one sample) were reported. We excluded peaks overlapping the ENCODE-defined blacklist regions (hg38). To address the variation in sequencing depths, across samples, we normalized sequencing depths per samples, and indirectly the resulting expression levels using random subsampling without replacement.^131^ The set of fragments (with lengths varying from 200 to 400 nts) *vs* the union-peaks were used to generate the ATAC-peak expression matrix. Additional quality controls include the assessment of nucleosome signatures and TSS enrichment analysis. We filtered the fragments with nucleosome signals < 4 and TSS enrichment levels > 2. Peak intensities were normalized using the term frequency inverse document frequency (TF-IDF) normalization (scale factor= 10,000). Samples from lines C3 and P3 were filtered according to the following criteria: number of reads between 3000 and 40000; number of features between 3000 and 30000; nucleosome signal below 0.75; TSS.enrichment score above 3; blacklist ration below 0.0025. For downstream analyses we used the Seurat^140^, Signac,^145^ and ArchR^146^ packages. The dimensionality reduction was performed using latent semantic indexing (LSI). Additionally, we performed Harmony integration across batches, which was used as input for the final clustering (resolution=0.2, SLM method^140^). Differentially expressed peaks were identified using the ‘findMarker’ function (Seurat package). We performed *de novo* motif analysis using Homer (*findMotifsGenome.pl*) and GO term enrichment analysis using GREAT with the background of the whole genome.^147^

#### Integrative analysis of multimaps profiles

The integration of snRNAseq and snATACseq signals was performed on 5,242 cells with matched barcodes. The crosstalk between modalities was assessed using the partitioning information obtained on single modalities. The co-variation in expressed was summarized in joint ATAC/RNA heatmaps, with Z scores, calculated per modality, on pseudobulked expression per gene being presented for the gene itself (RNA modality), TSS proximal peaks (<3kb) and TSS distal peaks (greater than 3kb and less than 50kb). Both ATAC and RNA modalities were used to infer regulons using SCENIC+.^148^

#### Re-analysis of *Kaufmann et al.*, *Lerma-Martin et al.*, and *Alsema et al.* spatial transcriptomics datasets

Count matrices with metadata annotations (when available) were downloaded for the following studies: 1) white matter Lerma-Martin et al.^84^ samples from UCSC Cell Browser, 2) white matter Alsema et al.^85^ samples under accession GSE208747, and 3) grey matter samples from Kaufmann et al.^86^ under accession GSE174647. Individual spatial datasets were converted into *Seurat* objects (v5.2.1). Spots containing >100 unique genes were kept for downstream analyses. The two white matter (WM) datasets were concatenated and analyzed separately from Kaufmann et al.^86^ grey matter (GM) dataset.

#### Spatially aware clustering and cell type deconvolution

For the Lerma-Martin et al.^84^ and Kaufmann et al.^86^ datasets the metadata were publicly available and used throughout our analyses. For the Alsema et al.^85^ dataset, the metadata needed to be calculated anew. Briefly, spatially aware clustering was performed using *BayesSpace* (v1.16)^127^ on a sample-by-sample basis, followed by spatial pre-processing (*nHVGs = 500, n.PCs = 15, log. normalize = T*); subsequently spatial clustering with optimized parameters (*q = 8 clusters, d = 15 PCs, init.method = mclust, model = t, nrep = 25000 MCMC iterations*) was applied. The spatial clusters were annotated according to their predicted tissue niches, i.e., lesion core, lesion rim, peri-lesional WM, normal-appearing WM, control WM, and GM; and compared with author-reported annotations, showing strong agreement. Cell type deconvolution was performed using the *spacexr* package (v2.2.1)^128^ implementing the Robust Cell Type Deconvolution (RCTD) algorithm. Using a pre-processed single-nuclei RNA sequencing dataset derived from samples of the identical tissue blocks used for the spatial dataset (Lerma-Martin et al.^84^) as a reference, RCTD was run in *full* mode.

#### Gene signature enrichment and DARG-high thresholding in spatial datasets

The localization of DARGs was assessed using a gene signature enrichment approach based on two methods: 1) *AUCell* (v1.28), and 2) a custom voting count scheme. When computing AUC scores, zero-count genes were discarded from building rankings (*keepZeroesAsNA = TRUE*). A permutation analysis using AUCell with 10,000 randomly sampled genes sets of length equal to the DARG gene signature set was conducted to infer the background distribution of AUC scores. The custom voting scheme relies on counting the number of signature genes expressed on a spot-individual basis. To conservatively estimate DARG presence, after careful inspection of DARG enrichment distributions across spatial niches and samples, ‘DARG-High’ spots were defined with DARG AUC scores above the confidence thresholds established by the AUCell permutation analysis, and simultaneously expressing >6 genes in WM samples, or >4 genes in GM samples from both radial glia and inflammatory DARG subsets. To mitigate the curse of dimensionality in statistical testing, enrichment scores within each grouping were quantile-binned, per bin averaged, and consequently used as input for an unpaired two-tailed *t*-test.

### Quantification and statistical analysis

For all phenotypic analyses, p-values were corrected using the Benjamini-Hochberg approach; adjusted p-value < 0.05 was considered significant (*). We performed statistical tests described in individual figure legends using Prism software version 10 (GraphPad Software, San Diego CA). For low throughput differential expression analysis on genes, we used a negative binomial test with the FDR cutoff value set to <0.05.

All analyses were performed on R 4.2.3, on a high memory computer (MacbookPro M1 Max, 64GB memory) and servers (Intel E7–8860v4, 3TB memory).

## Supplementary Material

Table S1

Table S2

Table S3

Table S4

Table S5

Table S6

Table S7

Table S8

9

SUPPLEMENTAL INFORMATION

Supplemental information can be found online at https://doi.org/10.1016/j.neuron.2025.09.022.

## Figures and Tables

**KEY RESOURCES TABLE:** 

REAGENT or RESOURCE	SOURCE	IDENTIFIER

Antibodies		

Mouse anti-OCT3/4 Mab	Santa Cruz	Cat#sc-5279; RRID:AB_628051
Rabbit anti-SOX2 PAb	Abcam	Cat#ab97959; RRID:AB_2341193
Chicken anti-NESTIN PAb	Novus Biolgicals	Cat#NB100-1604; RRID: AB_2282642
Rabbit anti-PAX6 PAb	Biolegend	Cat#901301; RRID:AB_2565003
Chicken anti-VIM PAb	Antibodies.com	Cat#A85421; RRID:AB_778824
Rabbit anti-Ki67 Mab	Abcam	Cat#ab16667; RRID:AB_302459
Alexa Fluor 546-conjugated Goat Anti Rabbit IgG	Thermo Fisher	Cat#A11010; RRID:AB_2534077
Alexa Fluor 488-conjugated Goat Anti Chicken IgY	Thermo Fisher	Cat#A11039; RRID:AB_2534096
Alexa Fluor 546-conjugated Donkey Anti Mouse IgG	Thermo Fisher	Cat#A10036; RRID:AB_11180613
Alexa Fluor 488-conjugated Donkey Anti Rabbit IgG	Thermo Fisher	Cat#A21206; RRID:AB_2535792
Mouse anti-p16^Ink4a^ Mab	Invitrogen	Cat#MA5-17093; RRID:AB_2538564
Rabbit anti-p21 Mab	Thermo Fisher	Cat#MA5-14949; RRID:AB_10984461
Rabbit anti-GDF15 PAb	Proteintech	Cat#27455-1-AP; RRID:AB_2880875
Mouse anti-b-actin Mab	Sigma	Cat# A1978; RRID:AB_476692
IRDye 680RD Goat anti-Rabbit	LI-COR Biosciences	Cat#926-68071; RRID:AB_10956166
IRDye 800CW Goat anti-Mouse	LI-COR Biosciences	Cat#926-32210; RRID:AB_621842

Biological samples		

Control fibroblasts (Table S1)	Ionescu et al.^18^ Douvaras et al.^111^	N/A
MS patient-derived fibroblasts (Table S1)	Ionescu et al.^18^ Douvaras et al.^111^	N/A

Chemicals, peptides, and recombinant proteins		

DMEM, high glucose, GlutaMAX	Thermo Fisher	Cat#61965026
Fetal bovine serum	Thermo Fisher	Cat#26140079
MEM Non-essential amino acids solution (100X)	Thermo Fisher	Cat#11140050
Sodium pyruvate (100 mM)	Thermo Fisher	Cat#11360070
Neurobasal Medium	Thermo Fisher	Cat#21103049
DMEM/F-12	Thermo Fisher	Cat#11320033
GlutaMAX Supplement	Thermo Fisher	Cat#35050038
B-27 Supplement (50X)	Thermo Fisher	Cat#17504001
N-2 Supplement (100X)	Thermo Fisher	Cat#17502001
CHIR99021	Cell Guidance Systems	Cat#SM13
Human LIF	PeproTech	Cat#300-05
SB-431542	Cayman Chemical	Cat#13031
StemMACS Y27632	Miltenyi Biotec	Cat#130-104-169
Growth Factor Reduced Basement Membrane Matrix	Corning	Cat#354230
Trypsin-EDTA (0.05%)	ThermoFisher	Cat#11580626
Accutase	Thermo Fisher	Cat#A1110501
Normal goat serum	Thermo Fisher	Cat#16-210-064
Normal donkey serum	Thermo Fisher	Cat# D9663
mTeSR Plus	STEMCELL Technologies	Cat#100-1130
hESC-Qualified Matrigel	Corning	Cat#354277
ReLeSR	STEMCELL Technologies	Cat#100-0483
DMEM/F-12, GlutaMAX supplement	Thermo Fisher	Cat#31331093
B-27 supplement, minus vitamin A	Thermo Fisher	Cat#12587001
Human Noggin recombinant protein	PeproTech	Cat#120-10C
Human FGF-basic (FGF-2)	PeproTech	Cat#100-18B
Laminin	Sigma-Aldrich	Cat#L2020
Poly-L-ornithine	Sigma-Aldrich	Cat#P4957
Human EGF	PeproTech	Cat#A-100-15
Fluoromount-G	Thermo Fisher	Cat#00-4958-02
DAPI fluorescence reagent for DNA	Thermo Fisher	Cat#D8417
DreamTaq Hot Start DNA Polymerase	Thermo Fisher	Cat#EP1702
dNTP Mix (10 mM each)	Thermo Fisher	Cat#R0192
Agarose	Scientific Laboratory Supplies	Cat#BIO41025
TAE buffer, 50X	MP Biomedicals	Cat#11TAE50X01
TrackIt 100bp DNA Ladder	Thermo Fisher	Cat#10488058
Gel Loading Dye (6X)	New England BioLabs	Cat#B7024S
GelRed Nucleic Acid Gel Stain	Biotium	Cat#41003
Navitoclax (ABT-263)	Selleck Chemicals	Cat#S1001
Fast SYBR Green Master Mix	Thermo Fisher	Cat#4385612
RIPA Lysis and Extraction Buffer	Abcam	Cat#ab156034
Halt Protease & Phosphatase Inhibitor Cocktail	Thermo Fisher	Cat#1861281
Prestained Protein Marker	Proteintech	Cat#PL00001
Intercept-TBS Blocking Buffer	LI-COR Biosciences	Cat#927-60001
NuPAGE LDS Sample Buffer (4X)	Invitrogen	Cat#NP0007
20X Bolt^™^ MOPS SDS Running Buffer	Invitrogen	Cat#B0001
Bolt^™^ Bis-Tris Plus Mini Protein Gels, 4–12%, 1.0 mm, WedgeWell^™^ format	Invitrogen	Cat# NW04122BOX
PVDF Transfer Membranes, 0.45 μm	Thermo Scientific	Cat#88518
Bovine Serum Albumin	Thermo Fisher	Cat#1002096887
Triton X-100	Thermo Fisher	Cat#1002575509
bisBenzimide H 33342 trihydrochloride (Hoechst)	Sigma-Aldrich	Cat#14533
eBioscience Propidium Iodide	Thermo Fisher	Cat#BMS500PI

Critical commercial assays		

DNeasy Blood & Tissue Kit	Qiagen	Cat#69506
RNeasy Mini Kit	Qiagen	Cat#74106
Pierce BCA Protein Assay Kit	Thermo Fisher	Cat#23227
Click-iT EdU Cell Proliferation Kit	Thermo Fisher	Cat#C10337
CytoTune-iPS 2.0 Sendai Reprogramming Kit	Thermo Fisher	Cat#A16517
Quant-iT PicoGreen dsDNA Assay Kits and dsDNA Reagents	Thermo Fisher	Cat#P7589
EZ DNA Methylation Gold Kit	Zymo	Cat#D5005
xGen Methyl-Seq Lib Prep 96rxn	Integrated DNA Technologies	Cat#1009824
Senescence Cell Detection (for microplate)	Dojindo	Cat#SG05-05
Cellular Senescence Detection Kit – SPiDER-β-gal	Dojindo	Cat#SG02-10
Human Cytokine Array C5	RayBiotech	Cat#AAH-CYT-5

Deposited data		

scRNAseq datasets	NCBI, GEO	GSE297365
snATACseq datasets	NCBI, GEO	GSE297690
WGBS datasets	NCBI, GEO	GSE251839
Bulk RNA-seq datasets	NCBI, GEO	GSE297192

Oligonucleotides		

Telo A, CGGTTTGTTTGGGTTTGGGTTTGGGTTTGGGTTTGGGTT	Custom primer from Sigma	N/A
Telo B, GGCTTGCCTTACCCTTACCCTTACCCTTACCCTTACCCT	Custom primer from Sigma	N/A
Hbg1, GCTTCTGACACAACTGTGTTCACTAGC	Custom primer from Sigma	N/A
Hbg2, CACCAACTTCATCCACGTTCACC	Custom primer from Sigma	N/A

Software and algorithms		

Adobe Illustrator	Adobe	RRID:SCR_010279
Fiji	GNU Generic Public License	RRID:SCR_002285
Prism	GraphPad	RRID:SCR_002798
FlowJo	BD	RRID:SCR_008520
CellProfiler	BSD	RRID:SCR_007358
Python	Python Programming Language	https://www.python.org
R	The R Project for Statistical Computing	https://www.r-project.org
fastQC	Babraham Bioinformatics	https://www.bioinformatics.babraham.ac.uk/projects/fastqc/
MultiQC	Ewels et al.^112^	https://multiqc.info/
noisyR	Moutsopoulos et al.^113^	https://core-bioinformatics.github.io/noisyR/
ClustAssess	Shahsavari et al.^114^	https://core-bioinformatics.github.io/ClustAssess/
STAR	Dobin et al.^115^	https://github.com/alexdobin/STAR
featureCounts	Liao et al.^116^	https://subread.sourceforge.net/
edgeR	Robinson et al.^117^	http://bioconductor.org/packages/release/bioc/html/edgeR.html
DESeq2	Love et al.^118^	https://github.com/mikelove/DESeq2
bulkAnalyseR	Moutsopoulos et al.^50^	https://github.com/Core-Bioinformatics/bulkAnalyseR
GENIE3	Huynh-Thu etal.^119^	https://github.com/aertslab/GENIE3
visNetwork	Almende et al.^120^	https://github.com/visjs/vis-network
CellRanger	10X genomics	https://www.10xgenomics.com
CellRanger ARC	10X genomics	https://www.10xgenomics.com
Seurat	Satija Lab	https://satijalab.org/seurat
Signac	Satija Lab	https://satijalab.org/signac
Harmony	Korsunsky et al.^121^	https://github.com/immunogenomics/harmony
Bismark	Krueger and Andrews^122^	https://www.bioinformatics.babraham.ac.uk/projects/bismark/
methylKit	Akalinetal.^123^	https://github.com/al2na/methylKit
Metilene	Juhling et al.^124^	http://legacy.bioinf.uni-leipzig.de/Software/metilene/
gProfiler	Kolberg et al.^125^	https://github.com/Granulate/gprofiler
AUCell	Aibar er al.^126^	https://www.bioconductor.org/packages/release/bioc/html/AUCell.html
BayesSpace	Zhao et al.^127^	https://www.bioconductor.org/packages/release/bioc/html/BayesSpace.html
RCTD (spacexr)	Cable et al.^128^	https://www.bioconductor.org/packages/release/bioc/html/spacexr.html
DARG Analysis Code (*in vitro* and post-mortem datasets)	This study	https://doi.org/10.5281/zenodo.15581507
Spatial Transcriptomics Re-analysis Code	This study	https://doi.org/10.5281/zenodo.15584699

## Highlights

- Human iNSCs maintain epigenetic age with high fidelity
- PMS fibroblasts and iNSCs share hypomethylated genes driving IFN signaling
- PMS iNSCs harbor senescent, IFN-responsive RG-like cells that promote inflammation
- Non-neurogenic senescent DARGs are found in lesions of people with PMS
