# Supplementary material for: Integrated omics reveals disease-associated radial glia-like cells with epigenetically dysregulated interferon response in multiple sclerosis
Source: Neuron. Author manuscript; Available in PMC 2026 Jan 26. (PMC12834227; doi:10.1016/j.neuron.2025.09.022)
Supplement: 9 [file NIHMS2136570-supplement-9.pdf]

Neuron, Volume *113*

Supplemental information

Integrated omics reveals disease-associated radial glia-like cells with epigenetically dysregulated interferon response in multiple sclerosis

Bongsoo Park, Alexandra M. Nicaise, Dimitrios Tsitsipatis, Liviu Pirvan, Daniel Zucha, Andi Munteanu, Pranathi Prasad, Miguel Larraz Lopez De Novales, Cristian Bulgaru, Rafael Kollyfas, Julia Whitten, Cory M. Willis, Luka Culig, Joseph Llewellyn, Rosana-Bristena Ionescu, Magdy Mekdad, Madalena B.C. Simões-Abade, Grzegorz Krzak, Jinshui Fan, Supriyo De, Matthew O. Ellis, Marta Suarez Cubero, Angeliki Spathopoulou, Luca Peruzzotti-Jametti, Tommaso Leonardi, Gabriel Balmus, Frank Edenhofer, Myriam Gorospe, Lukas Valihrach, Irina Mohorianu, Stefano Pluchino, and Isabel Beerman

SUPPLEMENTAL FIGURES

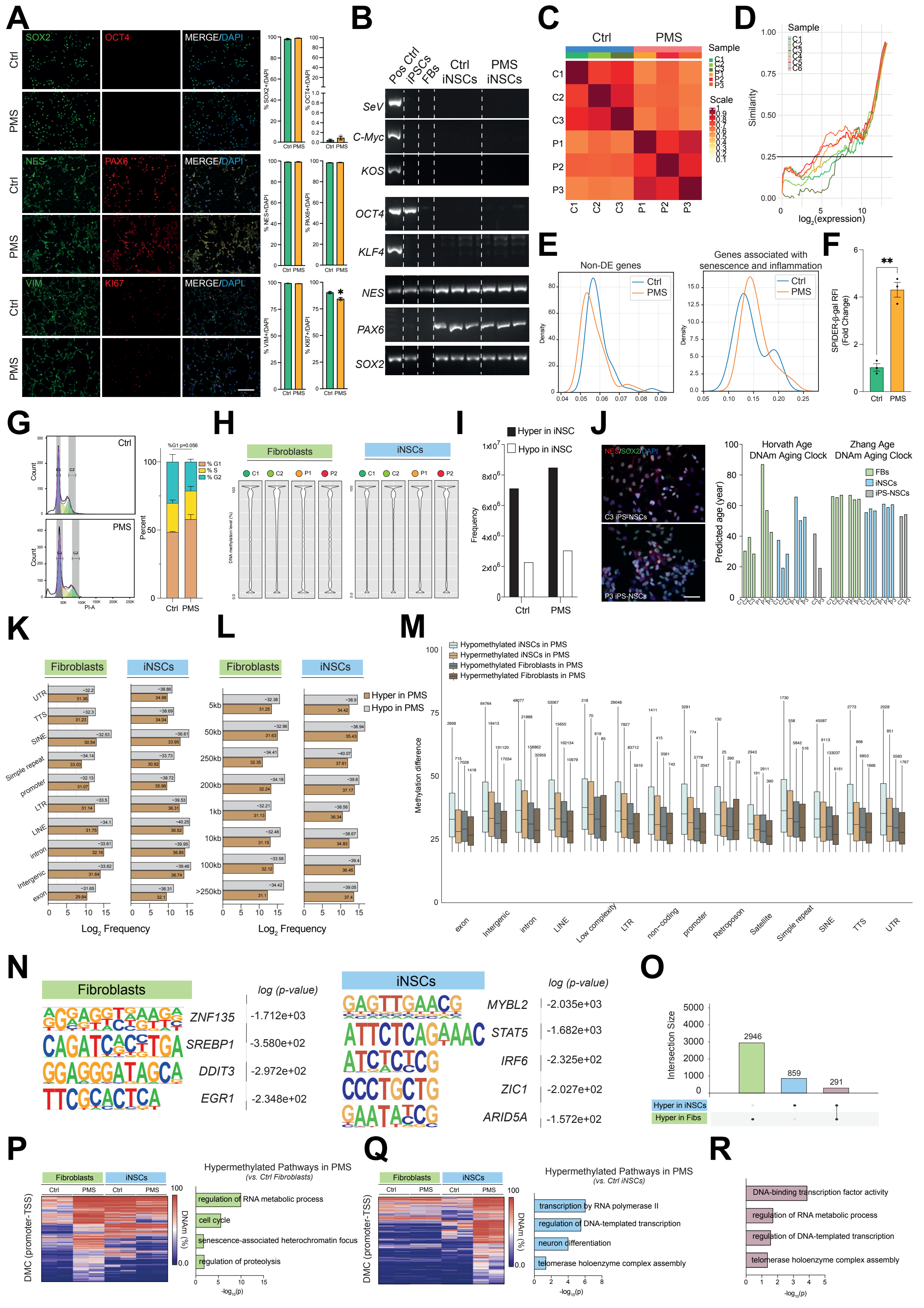

Figure S1. Supporting analyses on bulk RNA sequencing and whole-genome bisulfite sequencing (related to Figures 1 and 2)

(**A**) Immunocytochemistry and quantification of NSC markers. Each data point plotted is an individual cell line. Scale bar: 100 μm.

(**B**) RT-PCR gel of transgenes from Sendai virus reprogramming, pluripotency genes, and accepted NSC genes. Pos Ctrl, positive control; FBs, fibroblasts.

(**C**) Jaccard similarity index summarizing the experimental design of the bulk RNA-seq datasets.

(**D**) Identification of noise threshold using the noisyR framework.

(**E**) Density plots of the covariance in gene expression (derived from gene regulatory network (GRN) inference using GENIE3); networks inferred on Ctrl (blue) and PMS (red) samples, with top 50 edges considered were built on non-differentially expressed genes (left) and differentially expressed genes associated with senescence and inflammation (right).

(**F**) Quantification of relative fluorescence intensity (RFI) of senescence-associated β-galactosidase expression using SPiDER- β-gal. Data represented as a fold change over Ctrl iNSCs. Each data point plotted is an individual cell line.

(**G**) Flow cytometry-based quantification of iNSC cell cycle states and representative plots. Data plotted as cells in percent of cell cycle state.

(**H**) Summary of DNA methylation levels on fibroblast and iNSC samples.

(**I**) Frequency of differentially methylated cytosines (hyper and hypomethylated) in Ctrl and PMS cells, plotted as fibroblasts *vs* iNSCs.

(**J**) Representative immunocytochemistry of NSC markers in iPS-NSC lines and Horvath and Zhang predicted DNA methylation aging clock results on parental fibroblasts, iNSCs, and iPS-NSCs. Scale bar: 50 μm.

(**K**) Frequency of hyper and hypomethylation of PMS fibroblasts and iNSCs *vs* Ctrl based against gene annotation classes.

(**L**) Frequency of hyper and hypomethylation of PMS fibroblasts and iNSCs *vs* Ctrl based on distance to TSS.

(**M**) Hyper and hypomethylation differences across annotations classes for PMS fibroblasts and iNSCs *vs* Ctrl. Numbers represent identified DMRs.

(**N**) HOMER *de novo* motif enrichment analysis of PMS fibroblasts and iNSCs from hypomethylated regions in (**Fig. 2D**) representing unique motifs to PMS fibroblasts and iNSCs. The motifs are represented using proportional sequence logos. TF, transcription factor.

(**O**) UpSet plot of hypermethylated genes in fibroblasts and iNSCs within the 3k upstream of the transcription start site (TSS) - defined in this context as the promoter region.

(**P-Q**) Heatmap and enrichment analysis of downstream genes of hypermethylated DMCs specific to PMS fibroblasts *vs* Ctrl and PMS iNSCs *vs* Ctrl.

(**R**) Enrichment analysis of commonly hypermethylated genes of PMS fibroblasts and iNSCs *vs* Ctrl.

Experiments in **A** and **F-G** were done on n= 3 Ctrl and n= 3 PMS iNSC lines each performed in triplicate. Data in **A** and **F-G** are mean values ± SEM. *$p \leq 0.05$, **$p \leq 0.01$, un-paired t-test, with unequal variances.

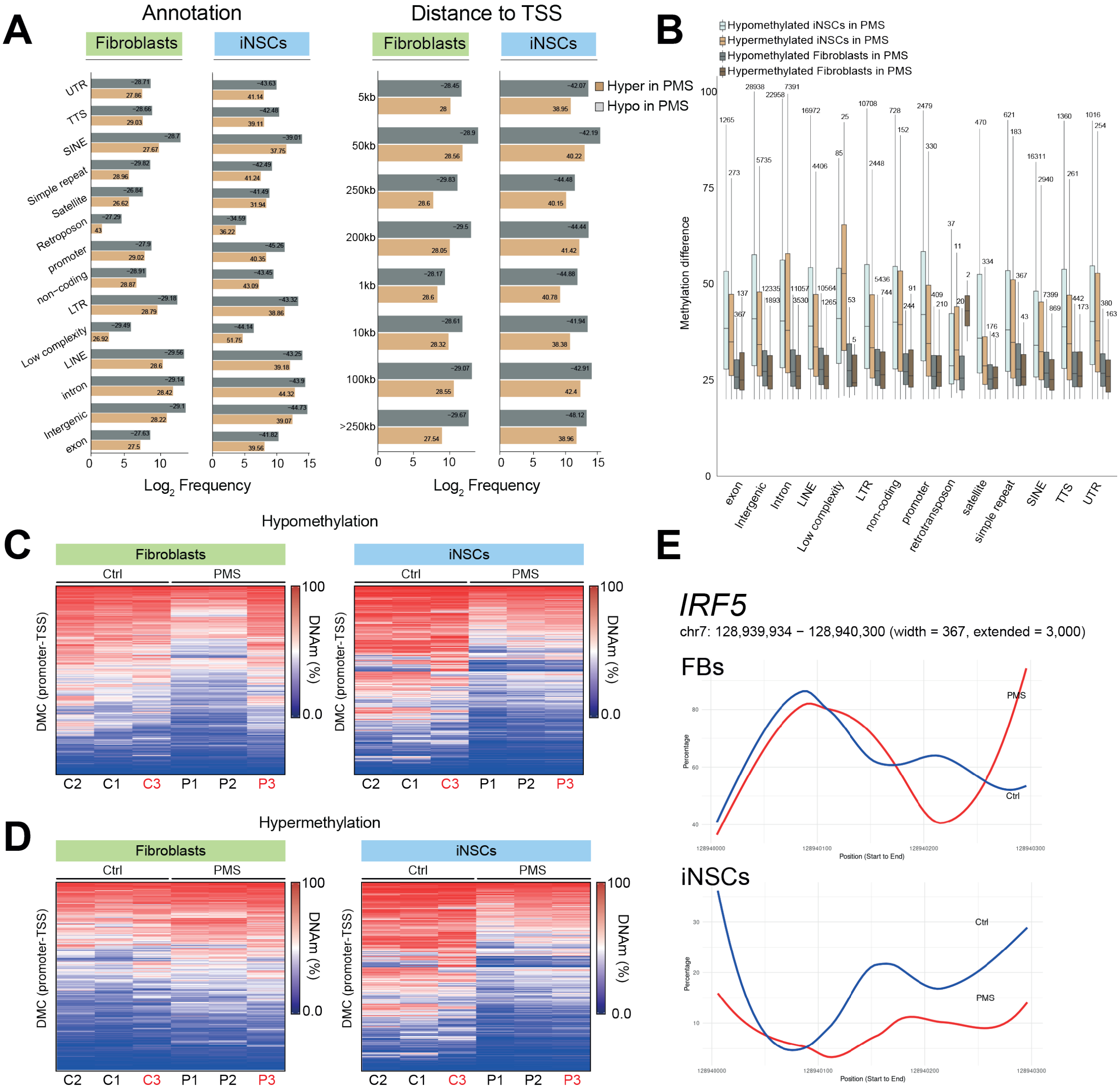

Figure S2. Additional lines (C3 and P3) recapitulate whole-genome bisulfite sequencing data (related to Figure 2)
(**A**) Frequency of hyper and hypomethylation of PMS fibroblasts and iNSCs *vs* Ctrl based against gene annotation classes and distance to TSS.
(**B**) Hyper and hypomethylation differences across annotations classes for PMS fibroblasts and iNSCs *vs* Ctrl. Numbers represent identified DMRs.
(**C-D**) Heatmap of hypomethylated and hypermethylated genes of all lines.
(**E**) Example of methylation difference for *IRF5* (genome browser tracks) in C3 and P3 lines.

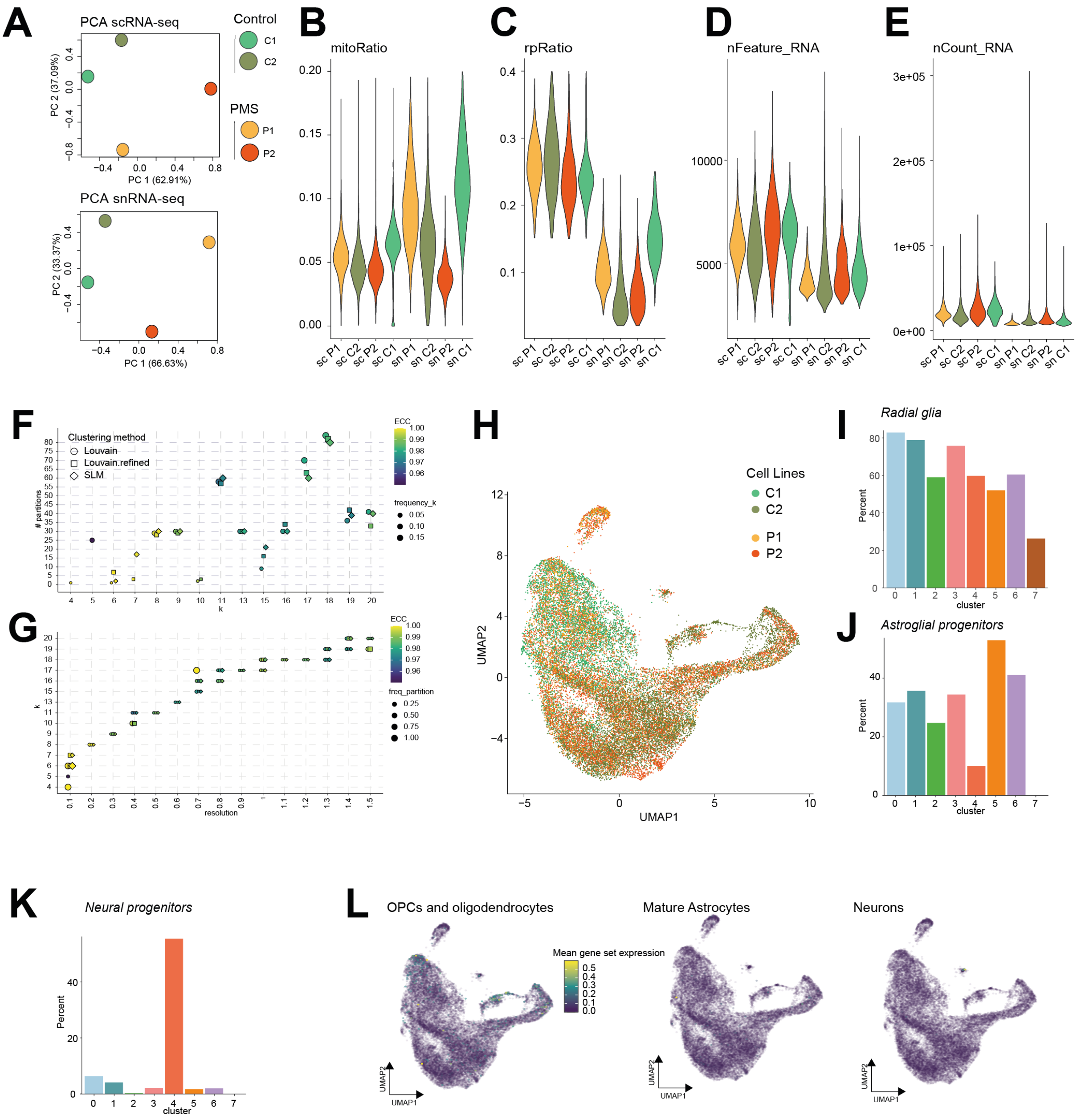

Figure S3. Quality control of sc/ snRNA sequencing dataset (related to Figure 3)
(**A**) PCA plots summarizing pseudobulked co-variation in expression of samples from iNSCs.
(**B**) Distribution of proportions of reads incident to mitochondrial genes per sample. A threshold of 20% was applied for filtering putative apoptotic cells.
(**C**) Distribution of proportion of reads incident to ribosomal proteins across samples; a threshold of 40% was applied; we also note a striking difference between single-cell and single-nuclei samples. MT and RP genes were excluded from downstream analyses prior to normalization.
(**D**) Distribution of nFeature (the number of genes per cell) in sc and snRNA-seq dataset.
(**E**) Distribution of nCount (the number of transcripts per cell) in sc and snRNA-seq dataset.
(**F**) ClustAssess summary figure illustrating the clustering stability, linking k (number of partitions correspondence with ECS threshold = 1, x-axis) *vs* the number of partitions (y-axis) derived from three clustering algorithms.
(**G**) ClustAssess summary figure illustrating cluster stability *vs* resolution (x-axis) and the number of clusters (y-axis).
(**H**) RNA UMAP illustrating the distribution of cells across the individual samples.
(**I-J**) Histograms representing percent of cells from individual cluster based on cell type, radial glia (**I**), astroglial progenitors (**J**), and neural progenitors (**K**).

(**L**) UMAPs of mean gene set expression for oligodendrocyte progenitor cells (OPCs) and oligodendrocytes, mature astrocytes, and neurons in the iNSCs.

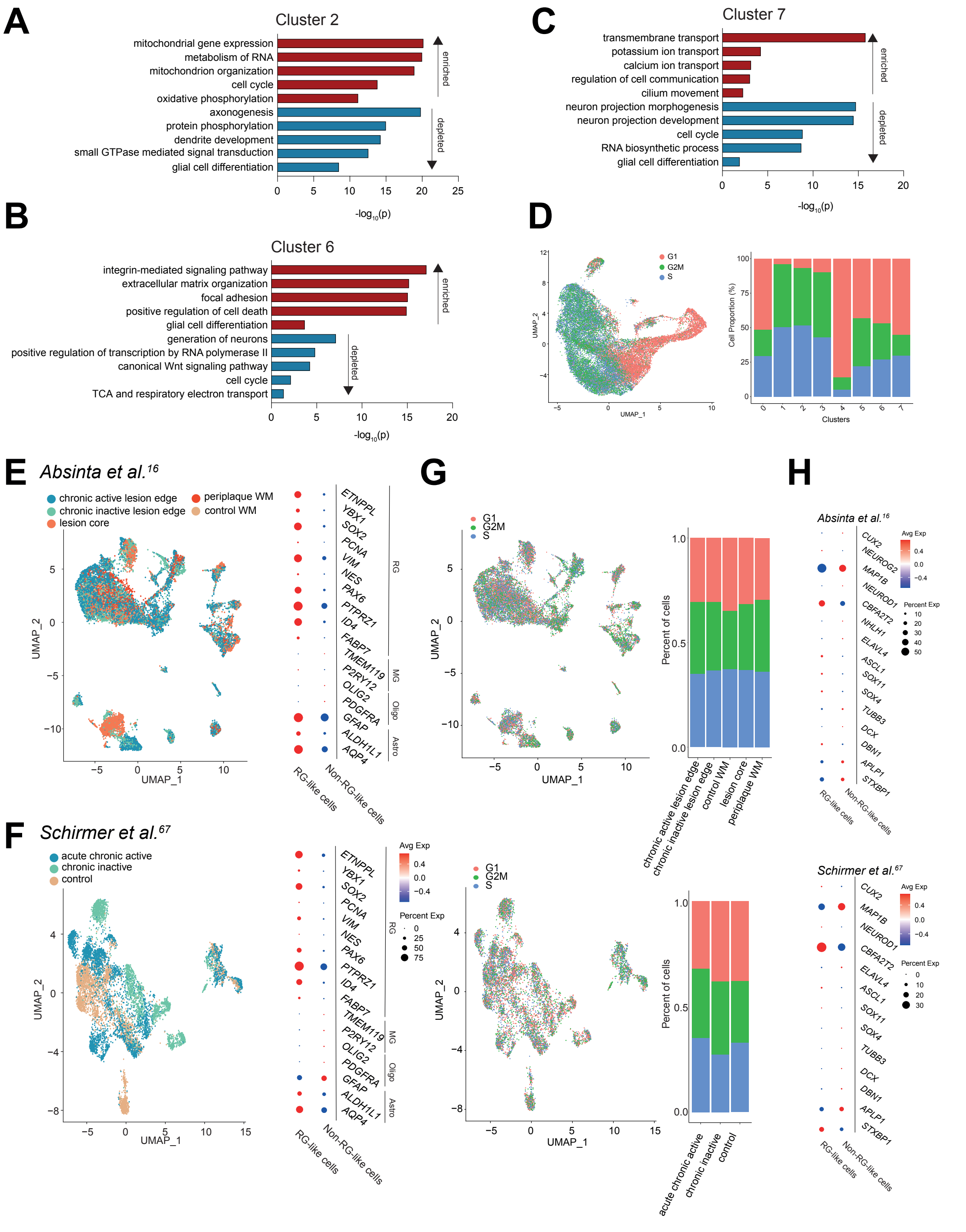

Figure S4. iNSCs do not contain differentiated cells and RG-like cells in the adult human brain are not neurogenic (related to Figure 3)

(A-C) Enrichment analysis depicting enriched and depleted terms in cluster 2, cluster 6, and cluster 7 *vs* all other clusters.

(**D**) UMAP summarizing cell cycle allocation (G1, G2M, and G) and stacked histogram summarizing proportions of cells per cluster assigned to each cell cycle phase.

(**E**, **F**) Recalculated UMAP based on RG gene expression displaying cells based on lesion location. Bubble plots displaying average expression and percent expression of RG markers, microglia (MG) markers, oligodendrocyte markers, and astrocyte markers in the identified DARGs and non-RG-like cells (**Fig. 3L-M**). WM, white matter.

(**G**) Bubble plots of neurogenic genes in RG-like and non-RG-like cells.

(**H**) UMAP summarizing cell cycle allocation and stacked histogram summarizing proportions of cells per area sequenced to each cell cycle state in the *ex vivo* data.

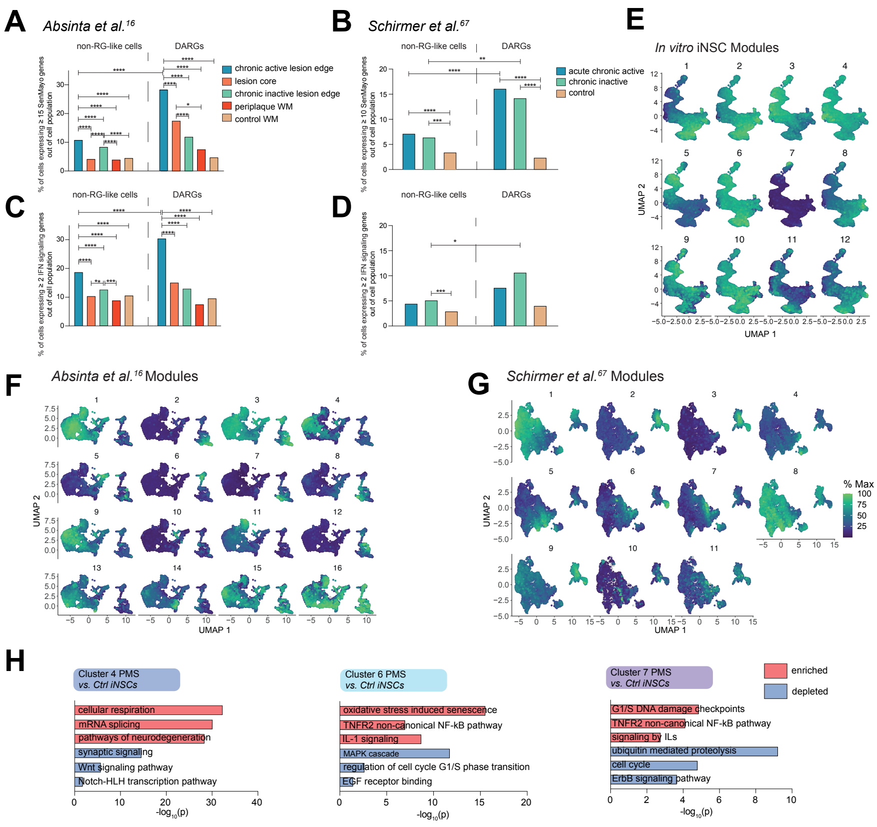

Figure S5. DARGs in chronic active lesion edges display senescence and IFN signaling genes (related to Figure 4 and 5)

(**A**) Relative proportion of cells expressing ≥ 15 SenMayo genes for DARGs and non-RG-like cells in the *Absinta et al.*[16] dataset, summarized per brain area sequenced.

(**B**) Relative proportion of cells expressing ≥ 10 SenMayo genes for DARGs and non-RG-like cells in the *Schirmer et al.*[67] dataset based on brain area sequenced.

(**C-D**) Relative proportion of cells expressing ≥ 2 IFN signaling genes for DARGs and non-RG-like cells in the *Absinta et al.*[16] (**C**) and *Schirmer et al.*[67] (**D**) dataset based on brain area sequenced.

(**E**) Gene modules generated on (dataset specific) inferred pseudotime from iNSC *in vitro* data.

(**F-G**) Gene modules generated on inferred pseudotime form *Absinta et al.*[16] and *Schirmer et al.*[67] recalculated UMAPs.

(**H**) Functional summary of enriched and depleted genes in cluster 4, cluster 6, and cluster 7 (Ctrl *vs* PMS, *in vitro* data).

Data in **A-D** are represented as percentages. Adjusted-p values, with a Benjamini-Hochberg multiple testing correction: *p≤ 0.05, **p≤ 0.01, ***p≤ 0.001, ****p≤ 0.0001 resulting from a Fisher's exact test on all pairwise comparisons.

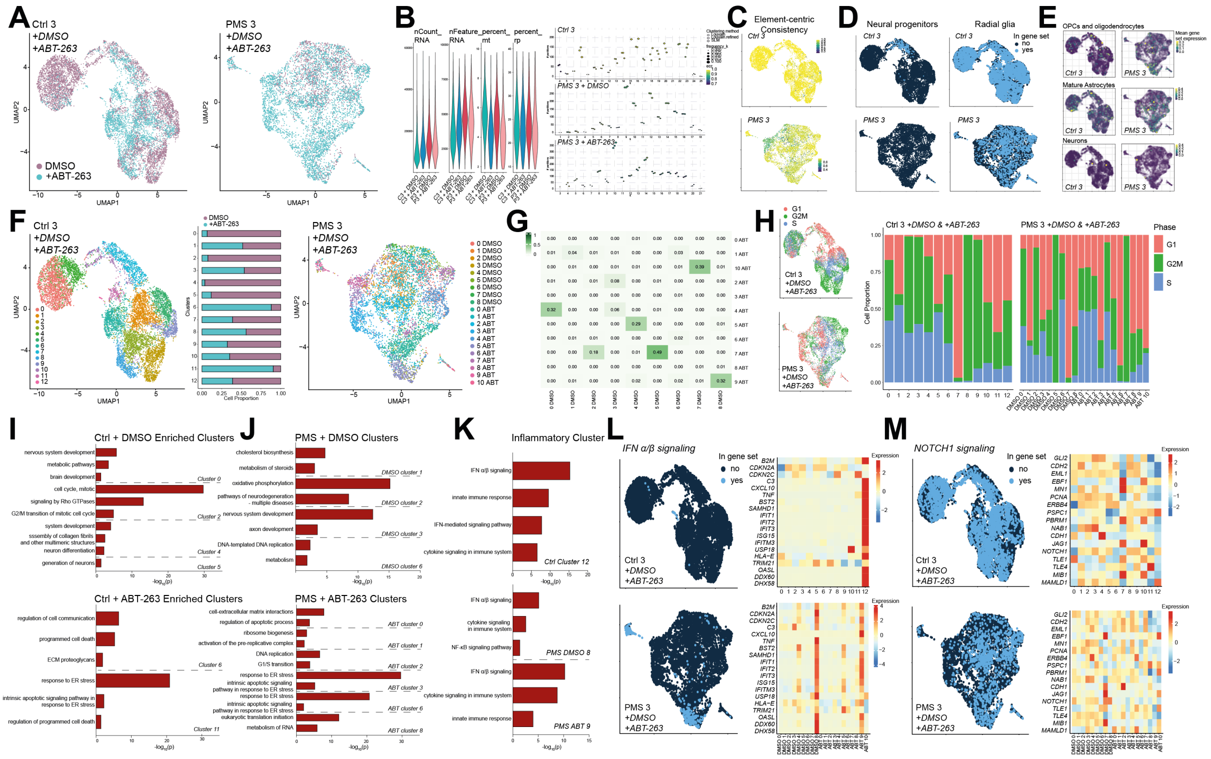

Figure S6. Treatment with senolytic ABT-263 reduces inflammatory and senescence signaling in PMS iNSCs (related to Figure 5)

(**A**) UMAPs of Ctrl line 3 (C3) and PMS line 3 (P3) single-cell RNA-seq samples.

(**B**) Distribution of nFeature and nCount in the scRNA-seq dataset. ClustAssess summary figure illustrating the clustering stability, linking k (number of partitions correspondence with ECS threshold = 1, x-axis) *vs* the number of partitions (y-axis) derived from three clustering algorithms.

(**C**) Element-centric consistency calculated per cell and visualized on the RNA UMAP.

(**D**) Voting scheme of genes associated with a neural progenitor and RG-like signature.

(**E**) UMAPs of mean gene set expression for oligodendrocyte progenitor cells (OPCs) and oligodendrocytes, mature astrocytes, and neurons.

(**F**) UMAPs of Ctrl and PMS samples (+ DMSO or + ABT-263) with individual clusters.

(**G**) Jaccard similarity index plot of PMS + DMSO and PMS + ABT-263 clusters.

(**H**) UMAP summarizing cell cycle allocation (G1, G2M, and G) and stacked histogram summarizing proportions of cells per cluster assigned to each cell cycle phase.

(**I**) Enrichment analysis depicting enriched terms in clusters enriched in the control + DMSO sample and Control + ABT-263 sample.

(**J**) Enrichment analysis depicting enriched terms in clusters enriched in the PMS + DMSO sample and PMS + ABT-263 sample.

(**K**) Enrichment analysis depicted enriched terms in the inflammatory iNSC cluster in both control and PMS iNSC samples.

(**L-M**) Heatmap of RNA inflammatory signature markers and summary of associated enrichment terms. Voting scheme UMAP of genes associated with IFN α/β signaling and NOTCH1 signaling.

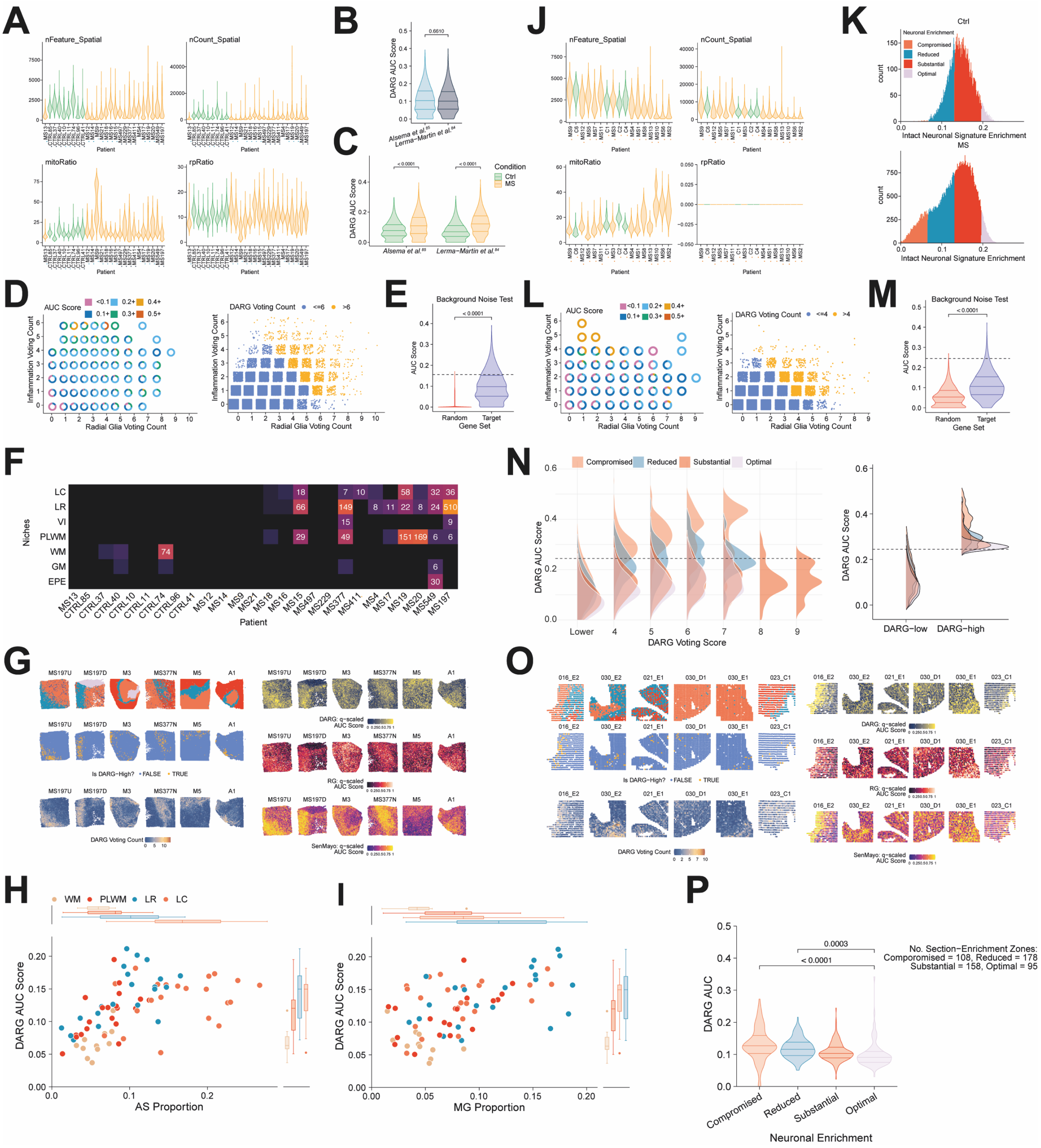

Figure S7 DARG enrichment in lesion-associated regions identified by spatial transcriptomics (related to Figure 7).
(**A** and **J**) Per-patient distribution of quality control metrics.
(**B**) DARG AUC Scores per study. Two-tailed unpaired *t*-test.
(**C**) DARG AUC Scores per condition and study. Two-tailed unpaired *t*-test.
(**D** and **L**) Relationship of DARG AUC Score with Radial Glia and Inflammation voting counts.
(**E** and **M**) Distribution of AUC Scores for DARG and randomly selected gene signatures (Target and Random, respectively). Random values are an assembly of 10,000 permutations for gene sets of equal length to DARG (16 genes).
(**F**) Number of DARG-High spots across niches in white matter samples.
(**G** and **O**) Exemplary white-matter (**G**) and grey-matter (**O**) sections, in the order of bottom-down left column first: niches (color-matched with panel F); DARG-High spots; DARG voting count; quantile-scaled DARG AUC Scores; quantile-scaled Radial Glia AUC Scores; quantile-scaled SenMayo Signature Scores.
(**H** and **I**) Relationship between astrocyte (AS) or microglia (MG) proportions and DARG AUC Score, color-coded by niches in white matter samples. A dot represents a niche's mean value per patient.

(**K**) Per-spot Intact Neuronal Signature Enrichment-dependent definition of spatial niches in the grey matter dataset.
(**N**) Color-coded by spatial niches, DARG AUC Score distribution across DARG voting counts and DARG-High spots, respectively.
(**P**) Distribution of DARG AUC Score for Neuronal Enrichment niches. Two tailed *t*-test with BH-correction.

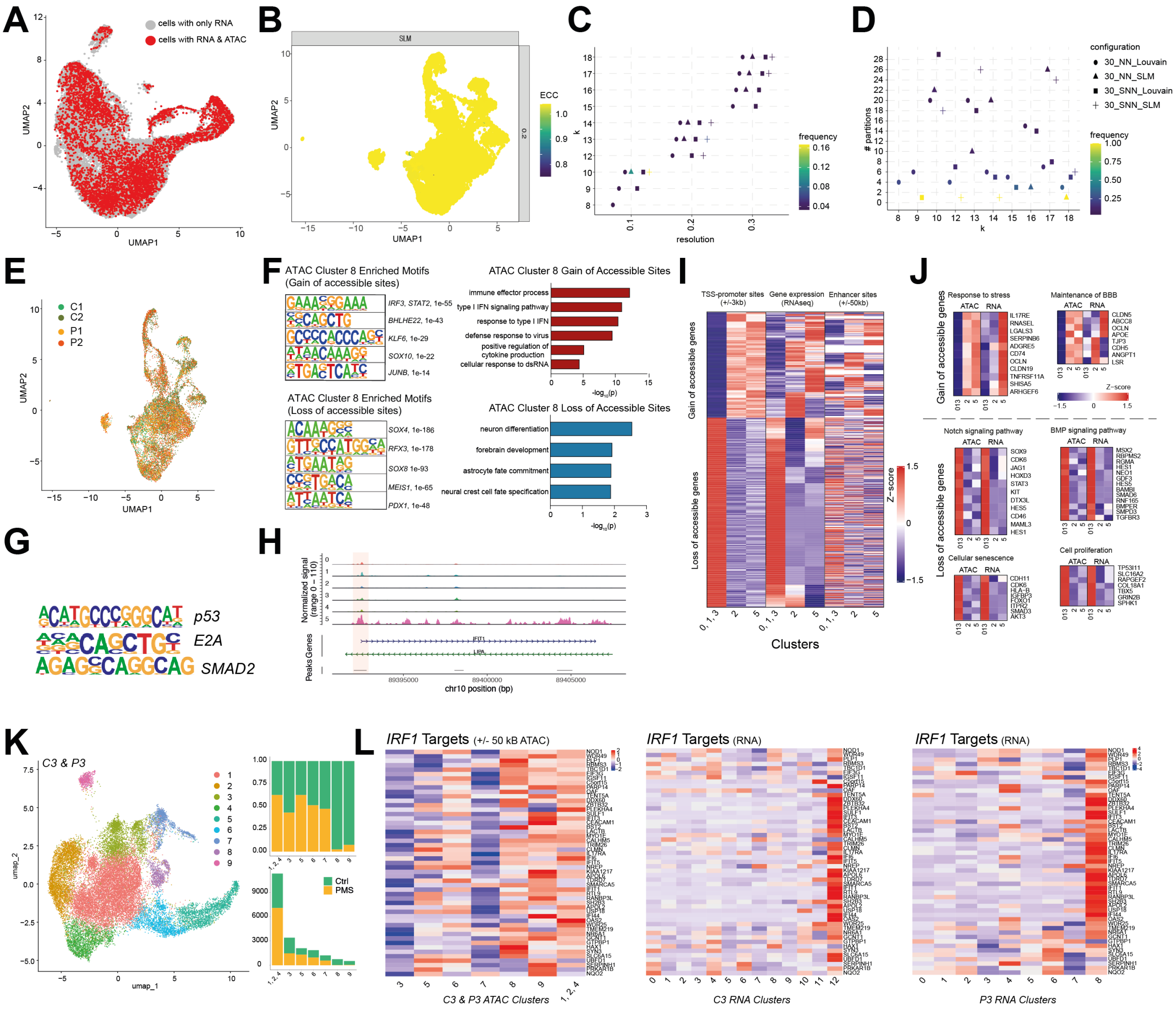

Figure S8. Summary of changes in chromatin accessibility specific to the inflammatory cluster. Assessment of functional variance on regulatory regions (related to Figure 8)
(**A**) UMAP of matched cells (red) with both RNA and ATAC expression and accessibility signatures, resulting from the multi-omics dataset.
(**B**) ClustAssess summary of ECC, represented on the ATAC UMAP, indicating a high stability of ATAC-centric clusters.
(**C-D**) Identification of stable, data-driven configurations for ATAC clusters, on 30 nearest neighbors (on weighted and unweighted neighborhoods) for variable resolution (**C**) and number of clusters (**D**).
(**E**) ATAC-centric UMAP summarizing the uniform distribution of cells across all samples.
(**F**) *De novo* motif analysis from gain of accessibility (GA) and loss of accessibility (LA) regions in ATAC cluster 8, summarized as proportional sequence logos; GREAT prediction of cis-regulatory regions using GA and LA regions in ATAC cluster 8.
(**G**) Summary of motifs, represented as proportional sequence logos, enriched in PMS fibroblasts and iNSCs on the WGBS modality and in cluster 8 via scATACseq.
(**H**) Genome browser tracks illustrating gain-of-accessible sites within *IFIT1* in RNA cluster 5.
(**I**) Heatmap summarizing differentially accessible regions (DARs) in 3kb regions upstream of the TSS. Heatmap includes the promoter region, the coding region, and enhancer sites for genes part of RNA-clusters 0,1,3, cluster 2, and cluster 5. The genes are sorted on loss/ gain of accessibility and on expression patterns.
(**J**) Dual heatmaps structured based on enrichment analyses, and allocation to pathways, summarizing the variation in expression and accessibility for genes with gain and loss of accessible sites, in RNA-clusters 2 and 5.
(**K**) UMAP of ATAC accessibility signatures in samples C3 and P3 (**Table S1**) and bar plots representing cluster proportion and number of cells in C3 and P3. Clusters 1, 2, and 4 are significantly enriched in PMS cells (X^2, 0.0127) and cluster 8 is enriched with control cells (X^2, 0.0416).

(**L**) Heatmaps of accessibility (ATAC) and expression (RNA) of *IRF1* targets in ATAC and RNA samples C3 and P3, as in **Fig. 8E**.
